# Catalytically active atomically thin cuprate with periodic Cu single sites

**DOI:** 10.1093/nsr/nwac100

**Published:** 2022-05-25

**Authors:** Huimin Yang, Shibo Xi, Na Guo, Mu Wang, Lingmei Liu, Pin Lyu, Xiaolong Yu, Jing Li, Haomin Xu, Xiao Hai, Zejun Li, Xinzhe Li, Tao Sun, Xiaoxu Zhao, Yu Han, Wei Yu, Jie Wu, Chun Zhang, Honghan Fei, Ming Joo Koh, Jiong Lu

**Affiliations:** Department of Chemistry, National University of Singapore, Singapore 117543, Singapore; Institute of Chemical and Engineering Sciences, Singapore 627833, Singapore; Department of Physics, National University of Singapore, Singapore 117542, Singapore; Department of Chemistry, National University of Singapore, Singapore 117543, Singapore; Advanced Membranes and Porous Materials Center, King Abdullah University of Science and Technology, Thuwal 23955-6900, Kingdom of Saudi Arabia; Department of Chemistry, National University of Singapore, Singapore 117543, Singapore; SDU-ANU Joint Science College, Shandong University, Weihai264209, China; Department of Chemistry, National University of Singapore, Singapore 117543, Singapore; Department of Chemistry, National University of Singapore, Singapore 117543, Singapore; Department of Chemistry, National University of Singapore, Singapore 117543, Singapore; Department of Chemistry, National University of Singapore, Singapore 117543, Singapore; Department of Chemistry, National University of Singapore, Singapore 117543, Singapore; Department of Chemistry, National University of Singapore, Singapore 117543, Singapore; School of Materials Science and Engineering, Peking University, Beijing 100871, China; Advanced Membranes and Porous Materials Center, King Abdullah University of Science and Technology, Thuwal 23955-6900, Kingdom of Saudi Arabia; Department of Chemistry, National University of Singapore, Singapore 117543, Singapore; Department of Chemistry, National University of Singapore, Singapore 117543, Singapore; Department of Chemistry, National University of Singapore, Singapore 117543, Singapore; Department of Physics, National University of Singapore, Singapore 117542, Singapore; Centre for Advanced 2D Materials and Graphene Research Centre, National University of Singapore, Singapore 117546, Singapore; Department of Chemistry, Tongji University, Shanghai200092, China; Department of Chemistry, National University of Singapore, Singapore 117543, Singapore; Department of Chemistry, National University of Singapore, Singapore 117543, Singapore; Centre for Advanced 2D Materials and Graphene Research Centre, National University of Singapore, Singapore 117546, Singapore

**Keywords:** exfoliation, nanosheet, heterogeneous catalysis

## Abstract

Rational design and synthesis of catalytically active two-dimensional (2D) materials with an abundance of atomically precise active sites in their basal planes remains a great challenge. Here, we report a ligand exchange strategy to exfoliate bulk [Cu_4_(OH)_6_][O_3_S(CH_2_)_4_SO_3_] cuprate crystals into atomically thin 2D cuprate layers ([Cu_2_(OH)_3_]^+^). The basal plane of 2D cuprate layers contains periodic arrays of accessible unsaturated Cu(II) single sites (2D-CuSSs), which are found to promote efficient oxidative Chan-Lam coupling. Our mechanistic studies reveal that the reactions proceed via coordinatively unsaturated CuO_4_(II) single sites with the formation of Cu(I) species in the rate-limiting step, as corroborated by both operando experimental and theoretical studies. The robust stability of 2D-CuSSs in both batch and continuous flow reactions, coupled with their recyclability and good performance in complex molecule derivatization, render 2D-CuSSs attractive catalyst candidates for broad utility in fine chemical synthesis.

## Teaser

A ligand exchange strategy exfoliates bulk cuprate crystals into atomically thin 2D cuprate layers with periodic unsaturated copper single sites, which are found to promote efficient oxidative Chan-Lam coupling.

## INTRODUCTION

Single-site catalysts (SSCs) or single-atom catalysts (SACs) with tunable local coordination environments of active sites have recently emerged as the frontier in heterogeneous catalysis due to their unique catalytic performance and maximized metal use efficiency [1–9]. However, achieving a high density of accessible single-metal active sites on the surface of solid supports remains difficult [10]. Two-dimensional (2D) materials with large surface areas have been actively explored in heterogeneous catalysis, but to enrich an abundance of catalytic sites in their basal planes is challenging [11–13]. Therefore, developing advanced 2D catalysts that marry the advantages of both SACs and 2D materials for efficient chemical transformations has significant fundamental interest and technological impact.

Copper-catalyzed Chan-Lam coupling is a key oxidative coupling process that is extensively used in organic synthesis for the production of pharmaceutically important aryl carbon-heteroatom compounds [14–17]. Homogeneous catalysts such as Cu(OAc)_2_ are predominantly employed in Chan-Lam coupling despite the problems of high metal loadings, non-reusability and laborious product separation/purification [18–20]. The synthesis of heterogeneous Cu catalysts by immobilizing Cu complexes on insoluble carriers or embedding Cu in three-dimensional frameworks has associated limitations of catalyst instability (e.g. metal leaching or clustering) or insufficient activity due to the formation of coordinatively saturated environments [21,22]. The major challenge lies in the development of solid-state catalytic materials with a high density of accessible unsaturated Cu coordination sites that remain active and stable under Chan-Lam coupling conditions.

Unlike Cu-containing layered double hydroxides (LDHs) with octahedral coordinatively saturated CuO_6_ motifs [23–27], layered cationic cuprate materials consist of positively charged inorganic layers with periodic arrays of coordinatively unsaturated Cu sites (including four-coordinated CuO_4_ sites with a square planar geometry and five-coordinated CuO_5_ sites with a tetragonal pyramid geometry), balanced by interlayer organic anionic ligands [28,29]. The weak electrostatic interaction between organic anions and cationic Cu hydroxide layers enables the delamination of bulk cuprate crystals into 2D atomic layers via a facile ligand exchange, whereby a desirably large number of coordinatively unsaturated Cu sites can be exposed for catalytic applications. To this end, we report a ligand exchange strategy for exfoliating layered cationic cuprate material [Cu_4_(OH)_6_][O_3_S(CH_2_)_4_SO_3_] into a new 2D cuprate catalyst ([Cu_2_(OH)_3_]^+^) containing periodic arrays of accessible coordinatively unsaturated Cu(II) single sites (2D-CuSSs). Because of their unique structure, these 2D-CuSSs are found to catalyze efficient Chan-Lam coupling. Both operando experimental and theoretical studies reveal that effective cross-coupling with 2D-CuSSs involves two adjacent coordinatively unsaturated single CuO_4_(II) sites with the generation of Cu(I) intermediate in the rate-determining step (see below for details).

## RESULTS AND DISCUSSION

### Synthesis and characterization of exfoliated 2D cuprate layers

**Figure 1.**
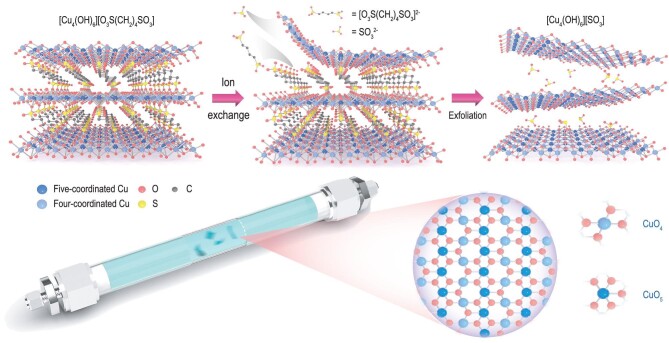
Schematic illustration of the synthesis of 2D-CuSSs. Illustration of the anion exchange strategy for the top-down exfoliation of bulk cuprate materials [Cu_4_(OH)_6_][O_3_S(CH_2_)_4_SO_3_] into atomically thin 2D-CuSSs, and atomic model of 2D-CuSSs. Note that dark blue, light blue, red, yellow and gray colorations represent CuO_5_, CuO_4_, O, S and C.

The TJU-1 crystals synthesized here consist of positively charged inorganic layers ([Cu_2_(OH)_3_]^+^) sandwiched between negatively charged 1,4-butanedisulfonate layers (Fig. 1), as confirmed by single-crystal X-ray diffraction [29]. Individual [Cu_2_(OH)_3_]^+^ layers are composed of periodic arrays of four-coordinated CuO_4_ sites (four intralayer Cu−O bonds), alternating with periodic arrays of five-coordinated CuO_5_ sites (five intralayer Cu−O bonds). Anion exchange has been widely used for the delamination of LDHs and other inorganic layered materials [30–32]. Unlike previous methods, our approach to exfoliating bulk TJU-1 materials relies on the exchange of the bidentate ligand with a monodentate one to prevent the exfoliated flakes from re-stacking. The electrostatic interaction between the α,ω-alkanedisulfonate anions and the cationic cuprate layers ensures that bulk TJU-1 can be exfoliated into atomically thin flakes, whereby the periodic arrays of under-coordinated CuO_4_ sites can be exposed to mediate organic transformations (Fig. 1). We therefore exploited the anion exchange strategy to exfoliate TJU-1 crystals in an aqueous solution of sulfite in ambient conditions. The weakly coordinating monodentate ligands can be easily dissociated from the open surface of 2D cuprate layers to facilitate chemical bond transformations. The resultant exfoliated nanosheets, namely 2D cuprate catalyst (2D-CuSSs), can be isolated as blue aggregates using centrifugation (Fig. S3).

**Figure 2.**
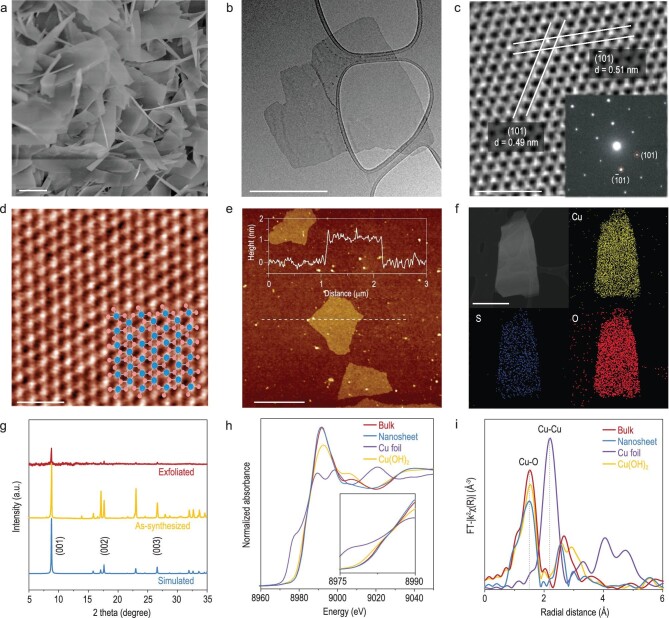
Characterization of 2D-CuSSs. (a) SEM image of the 2D-CuSSs. Scale bar: 1 μm. (b) TEM image of 2D-CuSS atomic layers. Scale bar: 300 nm. (c) Low-dose HRTEM lattice and selected area electron diffraction (SAED) of 2D-CuSSs. Scale bar: 2 nm. (d) iDPC-STEM image of the atomic lattice with the corresponding atomic model overlaid in the right corner. Scale bar: 1 nm. (e) AFM images and height profile of monolayer 2D-CuSSs. Scale bar: 1 μm. (f) TEM elemental mapping images of 2D-CuSS atomic layers. (g) XRD spectra of bulk cuprate materials (before exfoliation, yellow) and 2D-CuSSs (after exfoliation, red) and simulated pattern by SCXRD data (blue). (h) Cu K-edge XANES spectra and (i) the corresponding Fourier transformed EXAFS spectra of Cu foil (purple), commercial Cu(OH)_2_ (yellow), 2D-CuSSs before (blue) and after (red) exfoliation.

We then performed a series of measurements to characterize the structure of exfoliated 2D cuprate layers. Bulk TJU-1 crystals exhibit sharp (00h) peaks in the X-ray diffraction (XRD) spectrum, consistent with the simulated pattern (Fig. 2g). In contrast, the intensity of these features in the XRD spectrum of as-exfoliated flakes is significantly weakened. This suggests that most of the bulk crystals have been delaminated into individual nanosheets, as further confirmed by scanning electron microscopy (SEM) and transmission electron microscopy (TEM) imaging (Figs 2a and b and S1). An atomic force microscopy (AFM) image reveals a thickness of ∼1–2 nm for the majority of exfoliated nanosheets (∼80%), corresponding to 1–3 layers of exfoliated [Cu_2_(OH)_3_]^+^. The AFM height profile acquired over a few representative flakes (inset of Fig. 1e) reveals a thickness of ∼1 nm for monolayer exfoliated cuprate. It is noted that the value is slightly larger than the theoretical thickness of 6.5 Å expected for the monolayer cuprate, presumably arising from the physical corrugation of SiO_2_ substrate and the presence of weakly interacting sulfite anions on the surface. We also applied a shorter time for exfoliation to achieve the flakes with different thicknesses (Fig. S7).

Integrated differential phase contrast scanning transmission electron microscopy (iDPC-STEM) imaging resolves the periodic single Cu site arrays, consistent with the lattice model of a single cuprate layer (Fig. 2d). To reduce the possible beam damage, we also utilized low-dose high-resolution TEM to image the atomic lattice of exfoliated cuprate, resolving the lattice fringes along both (1 0 1) and (−1 0 1) basal planes (Fig. 2c). The atomic structure and coordination environment of Cu sites before and after exfoliation were further investigated by performing X-ray absorption fine structure (XAFS) analysis. It was observed that the valence state of Cu(II) and Cu−O bond length show a negligible change before and after exfoliation, as evidenced by X-ray photoelectron spectroscopy (XPS, Fig. S4), Cu K-edge X-ray absorption near edge structure (XANES, Fig. 2h) and Fourier transformed extended X-ray absorption fine structure (FT-EXAFS) spectra (Fig. 2i), respectively. Notably, the prominent features in the Cu K-edge FT-EXAFS spectra peaked at 1.51 Å and 1.50 Å for bulk crystals and 2D-CuSSs, respectively (corresponding to the intralayer Cu−O bond). All these observations indicate the successful exfoliation of bulk cationic TJU-1 crystals into atomically thin 2D-CuSS nanosheets, wherein the single-site Cu lattice in the cuprate layer remains after exfoliation.

### Examination of 2D-CuSSs in oxidative cross-coupling

Copper-mediated oxidative couplings have been widely used to enable aryl carbon-heteroatom bond formation, which is important for the fine chemical industry [14–17,33–36]. Cu(OAc)_2_ is commonly used as a general catalyst for this class of transformations. Compared to homogeneous catalysts, heterogeneous ones are much less explored even though they are arguably more attractive in the chemical industry due to their easier separation and recycling. Although a number of heterogeneous Cu catalysts have been reported for this reaction, problems such as low stability and/or metal leaching are common [37–41]. The 2D-CuSSs synthesized here contain a high density of accessible coordinatively unsaturated single Cu site arrays in 2D cuprate layers, which may potentially serve as efficient and robust heterogeneous catalyst candidates for cross-coupling reactions. Therefore, we first evaluated the catalytic performance of 2D-CuSSs in the oxidative Chan-Lam coupling between a variety of arylboronic acids and anilines. *p*-Tolylboronic acid and *p*-toluidine were utilized as model substrates to evaluate the conditions for Chan-Lam coupling, whereby 90% product yield could be obtained with 2D-CuSSs (Table 1, entry 11). In contrast, both bulk TJU-1 and commercial Cu(OH)_2_ powder that are deprived of open Cu(II) coordination sites were ineffective (entries 2–3). In addition, no reaction was observed with Cu-based MOF catalysts (HKUST-1) (entry 4), presumably due to the coordinatively saturated environment of Cu sites and tiny pore size (ca. 6 Å) that is presumably smaller than the substrate or product molecules. Although Cu-exchanged zeolite with a suitable pore size (∼1 nm) gave the coupling product a moderate yield (45%), appreciable leaching of ∼5% was detected even after one reaction cycle, as confirmed by inductively coupled plasma (ICP) analysis. The presence of 2,6-lutidine, acetic acid (10 mol%) and oxygen was crucial for efficient C−N coupling catalyzed by 2D-CuSSs (entries 7–9). Replacing acetic acid with sodium acetate was detrimental to the reaction (entry 10), highlighting the key role of the protic source. The thickness of the flakes had an impact on the yield of product (Table S4, entry 12). We further examined different protic donors in this catalytic transformation. Among the candidates evaluated, benzoic acid and myristic acid also delivered the desired product in 92% and 95% yield, respectively, whereas inorganic acids and phenol showed an inferior catalytic performance (<20% yield, Table S1). We chose acetic acid as the optimal acid additive throughout our study given its effectiveness and low cost.

**Table 1.** Optimization of reaction conditions with various copper-based catalysts.

		Cu	Acetic	
Entry	Catalyst	(mol%)	acid (mol%)	Yield^a^
1	-	0	10	0
2	Bulk	10	10	0
3	Cu(OH)_2_ + Na_2_SO_3_	10	10	0
4	HKUST-1 (MOF)	10	10	0
5	CU-Y (Zeolite)	10	10	45
6	Cu(OAc)_2_	10	10	71
7	2D-CuSSs^b^	10	10	11
8	2D-CuSSs^c^	10	10	43
9	2D-CuSSs	10	0	8
10	2D-CuSSs	10	0^d^	7
11	2D-CuSSs	10	10	90

^a^4-Methylphenylboronic acid (0.375 mmol, 1.5 equiv), *p*-toluidine (0.25 mmol, 1 equiv), acetic acid (0.025 mmol, 0.1 equiv), 2,6-lutidine (0.25 mmol, 1 equiv), toluene (1 mL), room temperature, air, 24 h, isolated yield based on ^1^H NMR analysis

^b^under N_2_

^c^no 2,6-lutidine

^d^NaOAc was used instead of AcOH.

**Figure 3.**
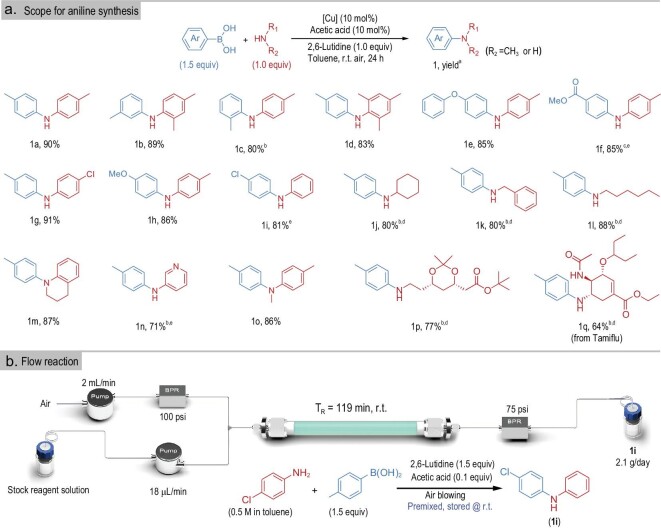
Substrate scope of 2D-CuSSs for C−N and C−O coupling. (a) 2D-CuSSs for C−N coupling. Reaction conditions for C−N and C−O coupling: aryl boronic acid (0.375 mmol, 1.5 equiv), amine or phenol (0.25 mmol, 1.0 equiv), catalyst (0.025 mmol), acetic acid (0.025 mmol), 2,6-lutidine (0.25 mmol, 1.0 equiv), toluene (1 mL), room temperature, 24 h. *^a^*Yield of corresponding amine and ether after reaction. Isolated yield. *^b^*CH_3_CN (1 mL) was used. *^c^*0.75 mmol boronic acid (3 equiv). *^d^*20% catalyst. *^e^*40ºC. (b) The schematic illustration of the flow reaction for 2D-CuSS catalyzed C−N coupling between *p*-tolylboronic acid and *p*-chloroaniline. BPR = back pressure regulator; psi = pounds per square inch.

We then tested a series of substituted anilines and boronic acids to examine the substrate scope of the C−N coupling catalyzed by 2D-CuSSs (Fig. 3a). The most porous catalysts (e.g. metal-organic frameworks (MOFs) and zeolites) suffer from diminished catalytic activity for substrates with relatively large molecular size (MW > 250). In stark contrast, 2D-CuSSs show good catalytic performance for compounds bearing a range of electronically and sterically diverse functional groups, affording the desired products in up to 91% yield (Fig. 3a). The C−N coupling transformations with 2D-CuSSs are also amenable to complex molecule derivatization. For instance, direct cross-coupling at the NH_2_ terminus of Tamiflu (antiviral medication) could be achieved to create **1q** at 64% yield. In contrast, the efficiency was much lower with the commonly utilized homogeneous catalyst Cu(OAc)_2_ (<1% yield; see Fig. S11), highlighting the distinct advantage of 2D-CuSSs over traditional homogeneous variants. This could be attributed to the large open surface of 2D-CuSSs with an abundance of Cu single sites (see below for further details).

2D-CuSSs also demonstrated high durability and recyclability for C−N coupling. 2D-CuSSs can be fully recycled after each run and the separation procedure ensures a negligible catalyst weight loss after each recycling run (Fig. S5). A TON > 450 can be readily achieved, higher than that of all the existing catalysts reported for this reaction [38,39,42–48]. A close examination reveals that the morphology and structure of 2D-CuSS nanosheets are retained (Figs S6 and S7) with only a trace amount of Cu, ∼0.5 ppm (<0.01% of the total Cu in the catalyst), present in the solution after each cycle (determined by ICP analysis).

As well as C−N coupling, 2D-CuSSs are also capable of promoting C−O coupling with a range of aryl boronic acids and phenols/alcohols under mild reaction conditions (Table S2). Notably, 2D-CuSSs offered good yields for C−O coupling, with two structurally sophisticated natural products, estradiol and cholesterol, whereas homogeneous Cu(OAc)_2_ was ineffective (<1% yield; see Fig. S12).

The utility of 2D-CuSSs in Chan-Lam coupling was further demonstrated by their application in continuous flow synthesis (Fig. 3b, S8, S9, S13, S14). A convenient home-made packed-bed reactor filled with 1.62 g of 2D-CuSSs and 0.5 g celite at each end (to avoid clogging) was assembled, which provided the C−N coupling product at 80% yield within a 2-hour residence time, leading to a production rate of 2.1 g/day. Compared to the 24-hour reaction time required in batch conditions, the significantly improved efficiency in the continuous flow mode can be attributed to the excellent gas/liquid/solid three-phase interaction and higher local concentration of the copper catalysts at a certain specific time. More importantly, the reaction yield remains nearly constant within 4 days of continuous flow production (note only a slight decrease of 4% in yield beyond 4 days), which highlights the robustness and practicality of 2D-CuSS catalysts.

### Mechanistic investigations involving 2D-CuSSs

**Figure 4.**
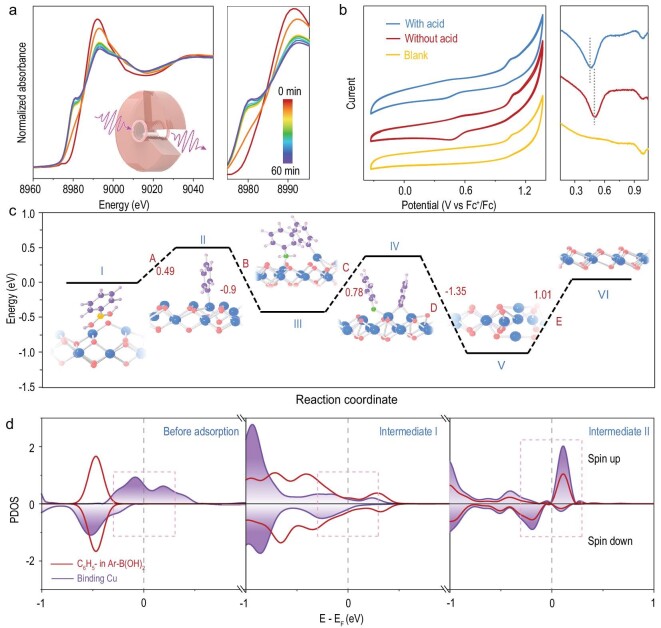
Probing the catalytic origin of 2D-CuSSs. (a) Operando Cu K-edge XANES spectra during the first hour of the C−N coupling between *p*-tolylboronic acid and *p*-toluidine. The inset illustrates the operando measurement set-up. (b) CV (left) and DPV (right) curves of 2D-CuSSs with acid and without acid treatment in toluene versus blank sample. (c) DFT-calculated reaction pathway for C−N coupling between *p*-tolylboronic acid and *p*-toluidine, together with the DFT-relaxed atomic structure of each intermediate (inset). Note that blue, red, yellow and gray colorations represent Cu, O, B and C respectively. (d) Partial density of states (PDOS) of the following reaction steps: before (panel I) and after (panel II) the pre-adsorption of phenylboronic acid on 2D-CuSSs via H bonding, and intermediate II (panel III), purple sphere and red solid line represent binding copper sites and phenyl group, respectively.

We then carried out density functional theory (DFT) calculations and operando XANES measurements to gain a better understanding of the origin of the catalytic performance of 2D-CuSSs. Inspired by the mechanistic insights derived from homogeneous Cu catalyst systems, we first considered a similar reaction pathway involving reactant adsorption and coupling over a single Cu site, which was found to be impossible due to the highly unfavorable steric hindrance. Given the challenges in DFT modeling associated with the heterogeneous nature of the catalyst, we only considered the most likely reaction pathway that involves the following steps as illustrated in Fig. 4c. An initiation process induced by the adsorption of phenylboronic acid over a CuO_4_ site through hydrogen bonding is followed by removal of one OH ligand on Cu (forming boric acid) to create sufficient space for the subsequent bond formation between phenyl and the Cu center (A, from Cu(II) to Cu(II)). Following transmetalation, aniline adsorbs on the adjacent CuO_4_ with a free coordination site (B, from Cu(II) to Cu(II)), followed by removal of one H from nitrogen (C, from Cu(II) to Cu(II)) in the presence of the lutidine base. Based on calculations, the adsorption of aniline on the same Cu center bearing phenyl was not feasible due to steric hindrance. The ensuing C−N bond formation across the two Cu sites creates the desired diphenylamine, which desorbs from 2D-CuSSs (D, from Cu(II) and Cu(II) to Cu(I) and Cu(I)). The integrity of the 2D-CuSSs is finally recovered by regaining one OH unit from the solution, presumably assisted by the presence of oxygen, amine and acetic acid [49] (E, from Cu(I) to Cu(II)). Amongst all the reaction steps, the calculated energy profile (Fig. 4c) reveals that steps A, C and E show an energy increase of 0.49, 0.78 and 1.01 eV respectively, while steps B and D result in energy decreasing by 0.9 and 1.35 eV, respectively. It is also expected that the coordination and valence state of Cu sites change as a reaction progresses. The Bader charge difference between 2D-CuSSs and intermediate V is determined to be ∼0.5 e^–^. The valence state of original Cu(II) recovers in the last step. According to the calculated energy profile, a barrier of higher than 1.01 eV needs to be overcome in the last step (the largest energy difference amongst all the steps). The intermediate state V is the most stable state in the reaction path shown in Fig. 4c. DFT calculations predict that the valence states of both Cu atoms participating in the reaction decrease by nearly 0.3 in the intermediate state V compared to the system before reaction. Therefore, we expect to observe a decrease in the valence state of Cu during the course of the reaction, consistent with our operando XANES measurements as will be discussed in a later section.

To probe the evolution of the catalyst electronic structure, we calculated the partial density of states (PDOS) of the phenyl group and the Cu atom at the reaction site with regard to the following steps (Fig. 4d): (i) before and (ii) after the adsorption of phenylboronic acid over 2D-CuSSs through hydrogen bonding, and (iii) the generation of intermediate II via transmetalation between phenylboronic acid and 2D-CuSSs. Before the adsorption of phenylboronic acid over the catalyst surface, there is negligible overlap of the PDOS between the phenyl group and Cu atom near Fermi level (*E*_F_, marked by dashed box in the left panel of Fig. 4d). The pre-adsorption of phenylboronic acid on 2D-CuSSs (intermediate I) induces significant hybridization between the phenyl group and Cu near *E*_F_ as can be seen from their overlapped PDOS (middle panel, Fig. 4d). This facilitates the electron transfer from Cu to phenyl in reaction step A to generate boric acid and intermediate II (phenyl group chemically bonds to Cu site), as evidenced by stronger hybridization between Cu and the phenyl group (reflected in the PDOS shown in the right panel, Fig. 4d). These results further suggest that the open four-coordinated Cu site favors electronic interaction with the phenyl group for the subsequent cross-coupling. Based on the analysis above, the large open surface of 2D-CuSSs, with an abundance of hydroxyl groups and active sites, also favors the adsorption of phenylboronic acid and sizeable amine molecules via hydrogen bonding, and thus facilitates their subsequent coupling. This highlights the unique advantage of 2D-CuSSs for sterically encumbered substrates, as compared to homogeneous Cu(OAc)_2_ wherein the steric hindrance may inhibit the coupling of sizeable reactants over a single Cu center.

To further corroborate the reaction mechanism proposed by DFT calculations, we performed the operando Cu K-edge XANES measurement to monitor the real-time evolution of the valence state of 2D-CuSSs as a function of reaction time. Within the first hour of the C−N coupling reaction between *p*-tolylboronic acid and *p*-toluidine, we observed that the peak at ∼8980 eV gradually arises, while the one at ∼8990 eV levels off (Fig. 4a). Moreover, the stationary phase after the first hour suggests a longer lifetime of Cu(I) species accumulated in the last step (Fig. S10). This is consistent with the proposed reaction mechanism, wherein Cu(I) species may survive for a longer period of time as the last step shows the largest energy barrier amongst all the reaction steps.

Another remaining question to address is the role of acetic acid, which is observed to be crucial for this system. Previous studies in homogeneous catalytic systems have shown that acetic acid can facilitate transmetalation by coordinating to the boron reagent [50]. Although this cannot be ruled out in our system, it is unclear whether such a pre-transmetalation process plays a role, since repeating the reaction with NaOAc instead of AcOH was inefficient (Table 1, entry 10). In other reports, it has been suggested that acetic acid facilitates the reoxidation of Cu(I) to Cu(II) in the reaction [49]. As shown in Fig. 4b, cyclic voltammetry (left) and differential pulse voltammetry (right) measurement reveals a reduction peak located at ∼0.4 V vs. Fc^+^/Fc for 2D-CuSSs. This reduction peak of 2D-CuSSs after acid treatment shifts to a more negative potential (0.45 V), indicating that Cu single sites in 2D-CuSSs are more prone to being oxidized in the presence of acetic acid. In addition, Cu K-edge XANES measurement reveals that the valence state of Cu in 2D-CuSSs after acid treatment increases, as compared to the sample without acid treatment (Fig. S11). Therefore, the presence of acetic acid is likely to assist the reoxidation of the Cu species [49,51], which facilitates the rate-determining step E from V to VI.

In conclusion, we demonstrated that a new 2D cuprate catalyst containing periodic arrays of accessible unsaturated Cu(II) single sites (2D-CuSSs) can be synthesized via top-down exfoliation through a well-designed ligand exchange strategy. 2D-CuSS atomic layers were found to be robust and efficient in catalyzing oxidative C−N and C−O couplings, with good recyclability. The presence of atomically well-defined active sites also allows us to explore structure–performance relationships and gain molecular-level insights into the reaction mechanism as revealed by both operando experimental and theoretical studies. Overall, the robust stability in both batch and continuous flow reactions, coupled with their good catalytic performance in the preparation of complex amine and ether compounds, underscores the potential applicability of 2D-CuSSs in fine chemical synthesis.

## METHODS

### Materials

Starting materials and solvents were purchased and used without further purification from commercial suppliers (Sigma-Aldrich, TCI, Adamas and others).

### Hydrothermal synthesis of TJU-1

A mixture of copper nitrate trihydrate (0.56 g, 2.5 mmol), 1,4-butanedisulfonate disodium salt (0.3 g, 1.2 mmol), cetyltrimethylammonium bromide (0.025 g, CTAB) and deionized water (8 mL) was added into a 15 mL Teflon-lined autoclave. The autoclave was then sealed and heated statically at 150°C for 3 days under autogenous pressure.

### Exfoliation of TJU-1 into Cu-SSCs

TJU-1 (31.8 mg, 10 mmol) was introduced to a 25 mL aqueous solution containing sodium sulfite (13 mg, 10 mmol). The mixture was then placed onto a shaker to shake continuously at 160 r/min for 24 h at ambient temperature. After 24 h, the mixture was centrifuged and the solids were washed with copious amounts of deionized water. Then the mixture was centrifuged at 500 r/min for 5 min to remove the unexfoliated TJU-1 particles. The supernatant was centrifuged at 12 000 r/min for 5 min to collect the Cu-SSCs as blue aggregates.

### Cu-catalyzed coupling of arylboronic acid and anilines

Solid arylboronic acid (0.375 mmol), 10 mol% (based on Cu) catalyst and 10 mol% of acetic acid were added into a 10 mL flask under an atmosphere of air, followed by the addition of 0.25 mmol 2,6-lutidine and 0.5 mL dry toluene using a syringe. The respective aniline (0.25 mmol) was then transferred to the solution, and the resulting mixture was shaken at ambient temperature for 24 h. After 24 h, the solution was concentrated, purified by column chromatography (silica gel) and analyzed by nuclear magnetic resonance (NMR) spectroscopy. To test recyclability, the Cu-SSCs were separated via centrifugation, washed with copious amounts of ethanol and dried under a vacuum. The dried nanosheets were used for the next round of catalysis for the same substrate.

### DFT calculations

The first-principles calculations were performed with spin-polarized DFT by utilizing the Vienna *ab-initio* Simulation Package (VASP) [52,53]. The generalized gradient approximation (GGA) in the Perdew-Burke-Ernzerhof (PBE) format and the projector-augmented wave (PAW) method were employed in all simulations with a plane wave basis, with a cut-off energy of 450 eV [54,55]. A vacuum layer of 15 Å was included to avoid the interaction with its mirror images. The convergence criteria for electronic steps and structural relaxations were set to 10-5 eV and 0.01 eV/Å, respectively.

The 3 × 3 supercell of 2D-CuSSs was adopted in this study, with a total of 36 Cu atoms, 54 O atoms and 54 H atoms. 2 × 2 × 1 K-sampling was applied in the calculations of both relaxation and DOS.

The thermal reaction energy diagram was calculated at 0 K and the Gibbs free energy diagram was calculated at 298 K. The Gibbs free energy was calculated as ΔG = ΔE + ΔZPE − TΔS, wherein zero-point energy (ZPE) is computed by the vibrational frequency. Temperature (T) was set to 298 K. The entropy of the gas-phase molecule was taken from the National Institute of Standards and Technology (NIST) database. In the frequency calculations, we only computed the vibrational frequencies of the adsorbates and underneath the Cu reaction center, while we kept other parts of 2d-CuSSs fixed.

## Supplementary Material

nwac100_Supplemental_File
