# Supplementary material for: Catalytically active atomically thin cuprate with periodic Cu single sites
Source: Natl Sci Rev. 2022 May 25;10(1):nwac100. doi: 10.1093/nsr/nwac100 (PMC9985158; doi:10.1093/nsr/nwac100)
Supplement: nwac100_Supplemental_File [file nwac100_supplemental_file.docx]

SUPPLEMENTARY INFORMATION

Catalytically Active Atomically Thin Cuprate with Periodic Cu Single Sites

Huimin Yang^1,9^, Shibo Xi^2,9^, Na Guo^3,9^, Mu Wang^1^, Lingmei Liu^4^, Pin Lyu^1^, Xiaolong Yu^5^, Jing Li^1^, Haomin Xu^1^, Xiao Hai^1^, Zejun Li^1^, Xinzhe Li^1^, Tao Sun^1^, Xiaoxu Zhao^6^, Yu Han^4^, Jie Wu^1^, Chun Zhang*^1,2,7^, Honghan Fei*^8^, Ming Joo Koh*^1^, Jiong Lu*^1,7^

^1^Department of Chemistry, National University of Singapore, Singapore 117543, Singapore.

^2^Institute of Chemical and Engineering Sciences, Singapore 627833, Singapore.

^3^Department of Physics, National University of Singapore, Singapore 117542, Singapore.

^4^Advanced Membranes and Porous Materials Center, King Abdullah University of Science and Technology, Thuwal 23955-6900, Kingdom of Saudi Arabia.

^5^SDU-ANU joint science college, Shandong University, Weihai, 264209, China.

^6^School of Materials Science and Engineering, Nanyang Technological University, Singapore, 639798, Singapore.

^7^Centre for Advanced 2D Materials and Graphene Research Centre, National University of Singapore, Singapore 117546, Singapore.

^8^Department of Chemistry, Tongji University, Shanghai 200092, China.

^9^These authors contributed equally

*E-mail: phyzc@nus.edu.sg; fei@tongji.edu.cn; chmkmj@nus.edu.sg; chmluj@nus.edu.sg

**1. General Procedure for flow reaction**

The micro-tubing reactor was packed by 1/4" OD x 0.188" ID of 27.3 cm PFA tubing. The stainless-steel beads were packed in PFA tubing. Residence times were calculated by dividing the free volume within the reactor by the liquid flow rates. A back-pressure regulator (100 psi) was used to achieve steady flow.

**2. General Procedure for products purification**

**tert-butyl-2-((4S,6S)-2,2-dimethyl-6-(2-(p-tolylamino)ethyl)-1,3-dioxan-4-yl)acetate (1p)**: 4-methylboronic acid (51.0 mg, 0.375 mmol), 2,6-lutidine (44 μL), acetic acid (2 μL) and 2D-CuSSs (10 mg) were mixed in dried Acetonitrile (2 mL). 1,1-Dimethylethyl(4S,6S)-6-(2-aminoethyl)-2,2-dimethyl-1,3-dioxane-4-acetate (69.0 mg, 0.25 mmol) was added to the mixture. The solution was then stirred at room temperature under air for 24 h. After removing the solvent under vacuum, dichloromethane was added and the mixture was washed using dilute aqueous NaCl solution, and then dried over Na_2_SO_4_, and concentrated in vacuo. The product was purified by column chromatography (silica gel, hexanes:EtOAc = 4:1) to afford 1p (67.2 mg, 0.19 mmol, 77% yield) as colorless oil. 1H NMR (400 MHz, CDCl3): δ 6.98 (d, 2H), 6.54 (d, 2H), 4.25 (q, 1H), 3.21 (m, 2H), 2.45-2.32 (m, 2H), 2.29 (s, 3H), 1.76 (m, 2H), 1.54 (d, 1H), 1.44 (s, 12H), 1.40 (s, 3H), 1.28-1.25 (m, 2H); 13C NMR (101 MHz, CDCl3): δ 170.08, 145.94, 129.54, 126.39, 112.94, 98.58, 80.47, 67.53, 66.05, 42.50, 41.12, 36.22, 35.36, 29.98, 20.20, 19.60; HRMS (EI) [M]+ calcd for C_21_H_34_NO_4_: 364.2482, found: 364.2490.

**ethyl-(3R,4R,5S)-4-acetamido-3-(pentan-3-yloxy)-5-(p-tolylamino)cyclohex-1-ene-1-carboxylate (1q)**: 4-methylboronic acid (51.0 mg, 0.375 mmol), 2,6-lutidine (44 μL), acetic acid (2 μL) and 2D-CuSSs (10 mg) were mixed in dried Acetonitrile (2 mL). Ethyl(3R,4R,5S)-5-amino-4-acetamido-3-(pentan-3-yloxy)cyclohex-1-ene-1-carboxylate (80.0 mg, 0.25 mmol) in dried Acetonitrile was added to the mixture. The solution was then stirred at room temperature under air for 24 h. After removing the solvent under vacuum, dichloromethane was added and the mixture was washed using dilute aqueous NaCl solution, dried over Na_2_SO_4_, and concentrated in vacuo. The product was purified by column chromatography (silica gel, hexanes:EtOAc = 4:1) to afford 1q (59.1 mg, 0.16 mmol, 64% yield) as colorless solid. 1H NMR (400 MHz, CDCl3): δ 6.95 (d, 2H), 6.84 (s, 1H), 6.48 (d, 2H), 4.22-4.17 (m, 3H), 4.04 (s, 1H), 3.68-3.66 (m, 1H), 3.42-3.39 (m, 1H), 2.83-2.78 (m, 1H), 2.41-2.35 (m, 1H), 2.21 (s, 3H), 1.88 (s, 3H), 1.55-1.50 (m, 4H), 1.30-1.26 (m, 4H), 0.93-0.87 (m, 6H); 13C NMR (101 MHz, CDCl3): δ 171.10, 166.33, 144.85, 136.29, 129.83, 126.46, 113.76, 113.02, 81.91, 75.14, 60.98, 53.72, 52.09, 30.22, 26.17, 23.38, 20.33, 14.20, 9.40; HRMS (EI) [M]+ calcd for C_23_H_34_N_2_O_4_: 403.2591, found: 403.2597.

**(8R,9S,13S,14S,17S)-13-methyl-3-(p-tolyloxy)-7,8,9,11,12,13,14,15,16,17-decahydro-6H-cyclopenta[a]phenanthren-17-ol (2j)**: 4-methylboronic acid (51.0 mg, 0.375 mmol), 2,6-lutidine (44 μL), acetic acid (2 μL) and 2D-CuSSs (10 mg) were mixed in dried Toluene (2 mL). Ethyl(3R,4R,5S)-5-amino-4-acetamido-3-(pentan-3-yloxy)cyclohex-1-ene-1-carboxylate (80.0 mg, 0.25 mmol) was added to the mixture. The solution was then stirred at room temperature under air for 24 h. After removing the solvent under vacuum, dichloromethane was added and the mixture was washed using dilute aqueous NaCl solution, dried over Na_2_SO_4_, and concentrated in vacuo. The product was purified by column chromatography (silica gel, hexanes:EtOAc = 5:1) to afford 2j (52.6 mg, 0.16 mmol, 64% yield) as colorless oil. 1H NMR (400 MHz, CDCl3): δ 7.25 (d, 1H), 7.16 (d, 2H), 6.86 (d, 2H), 6.70 (d, 1H), 6.33 (d, 1H), 5.75 (s, 1H), 4.50 (d, 1H), 3.54-3.51 (m, 1H), 2.73 (t, 2H), 2.27 (s, 4H), 2.14 (m, 1H), 1.86-1.77 (m, 3H), 1.59-1.57 (m, 1H), 1.38-1.23 (m, 6H), 0.67 (s, 3H); 13C NMR (101 MHz, CDCl3): δ 155.23, 138.59, 135.41, 130.75, 127.13, 119.05, 118.45, 116.03, 80.49, 50.02, 44.08, 43.26, 38.85, 37.03, 30.37, 29.54, 27.18, 26.42, 23.24, 20.71, 11.71.

**(3S,8S,9S,10R,13R,14S,17R)-10,13-dimethyl-17-((R)-6-methylheptan-2-yl)-3-(p-tolyloxy)-2,3,4,7,8,9,10,11,12,13,14,15,16,17-tetradecahydro-1H-cyclopenta[a]phenanthrene (2k)**: 4-methylboronic acid (51.0 mg, 0.375 mmol), 2,6-lutidine (44 μL), acetic acid (2 μL) and 2D-CuSSs (10 mg) were mixed in dried Toluene (2 mL). (3β)-Cholest-5-en-3-ol (97.0 mg, 0.25 mmol) was added to the mixture. The solution was then stirred at room temperature under air for 24 h. After removing the solvent under vacuum, dichloromethane was added and the mixture was washed using dilute aqueous NaCl solution, dried over Na_2_SO_4_, and concentrated in vacuo. The product was purified by column chromatography (silica gel, hexanes:EtOAc = 4:1) to afford 2k (56.1 mg, 0.12 mmol, 51% yield) as colorless solid. 1H NMR (400 MHz, CDCl3): δ 7.02 (d, 2H), 6.74 (d, 2H), 5.35 (d, 1H), 3.56-3.50 (m, 1H), 2.27 (s, 5H), 1.99-1.82 (m, 6H), 1..50-1.25 (m, 11H), 1.12-1.01 (m, 11H), 0.92-0.86 (m, 10H), 0.68 (s, 3H); 13C NMR (101 MHz, CDCl3): δ 156.95, 153.78, 140.87, 130.14, 121.88, 115.27, 72.00, 56.92, 56.31, 50.28, 42.47, 39.94, 39.67, 37.40, 36.65, 36.34, 35.94, 32.06, 31.79, 28.38, 28.16, 24.44, 23.98, 22.96, 22.71, 21.24, 20.60, 19.54, 18.87, 12.01.

**3. The characterization of as-prepared catalysts.**

Low-dose HRTEM experiments were carried on a Cs-corrected FEI cubed G2 Titan 60-300 electron microscope operated at 300 kV, with a Gatan K2 direct-detection camera. The images were acquired in electron counting mode with the dose fractionation function. Integrated differential phase contrast scanning transmission electron microscopy (iDPC-STEM) was performed on a double Cs-corrected electron microscope (FEI Titan Cubed Themis Z) operated at 300 kV. A high-pass filter was applied to reduce the low-frequency noises. 1H and 13C NMR spectra were recorded on either Bruker AV500 (500 MHz) or AVIII400 (400 MHz) spectrometer. Chemical shifts were reported in parts per million (ppm), and the residual solvent peak was used as an internal reference: 1H (chloroform δ 7.26; DMSO δ 2.50), 13C (chloroform δ 77.0; DMSO δ 40). The extended X-ray absorption fine structure (EXAFS) measurements of Cu K-edge were carried out at the XAFCA beam line of the Singapore Synchrotron Light Source (SSLS). The storage ring of SSLS operated at 700 MeV with beam current of 250 mA. A Si (111) double-crystal monochromator was applied to filter the X-ray beam. Cu foils were used for the energy calibration, and all samples were measured under transmission mode at room temperature. The EXAFS oscillations χ(k) were extracted and analyzed using the Demeter software package.

**Supplementary Figures**


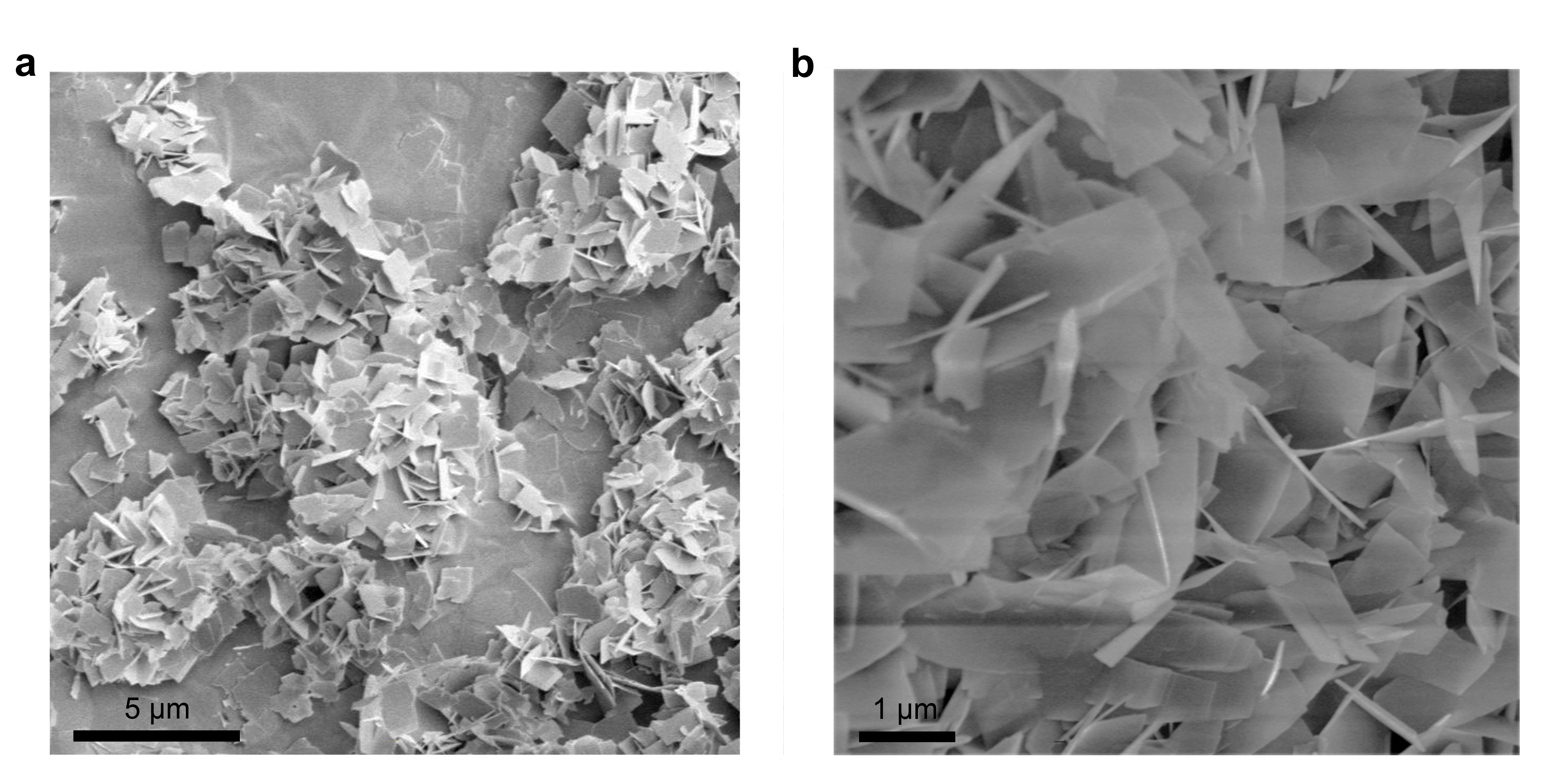


**Supplementary Fig. 1**: SEM images of 2D-CuSSs.


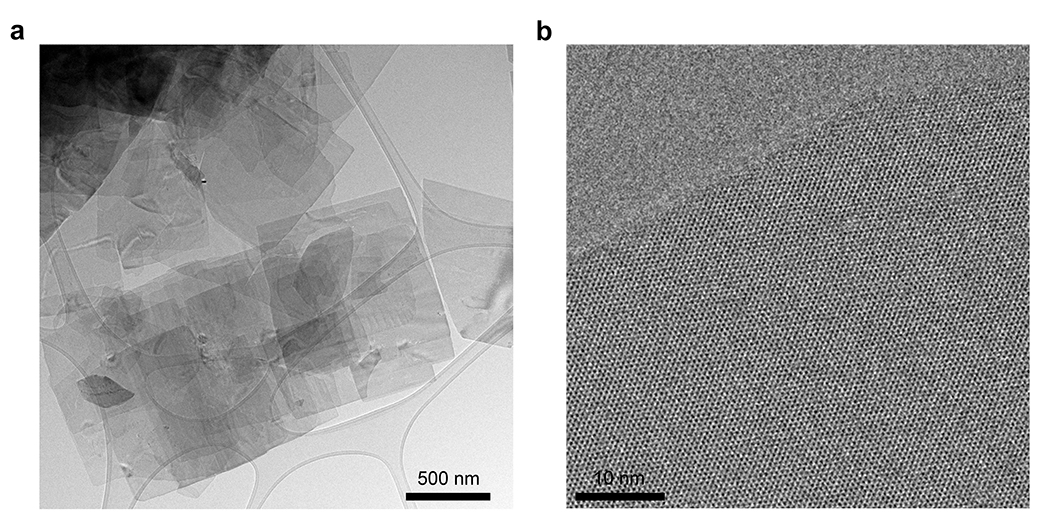


**Supplementary Fig. 2**: TEM (a) and HRTEM (b) images for 2D-CuSSs (a) Large scale of TEM image of 2D-CuSSs. Scale bar: 500 nm; (b) Large scale Low-dose HRTEM image of 2D-CuSSs. Scale bar: 10 nm.


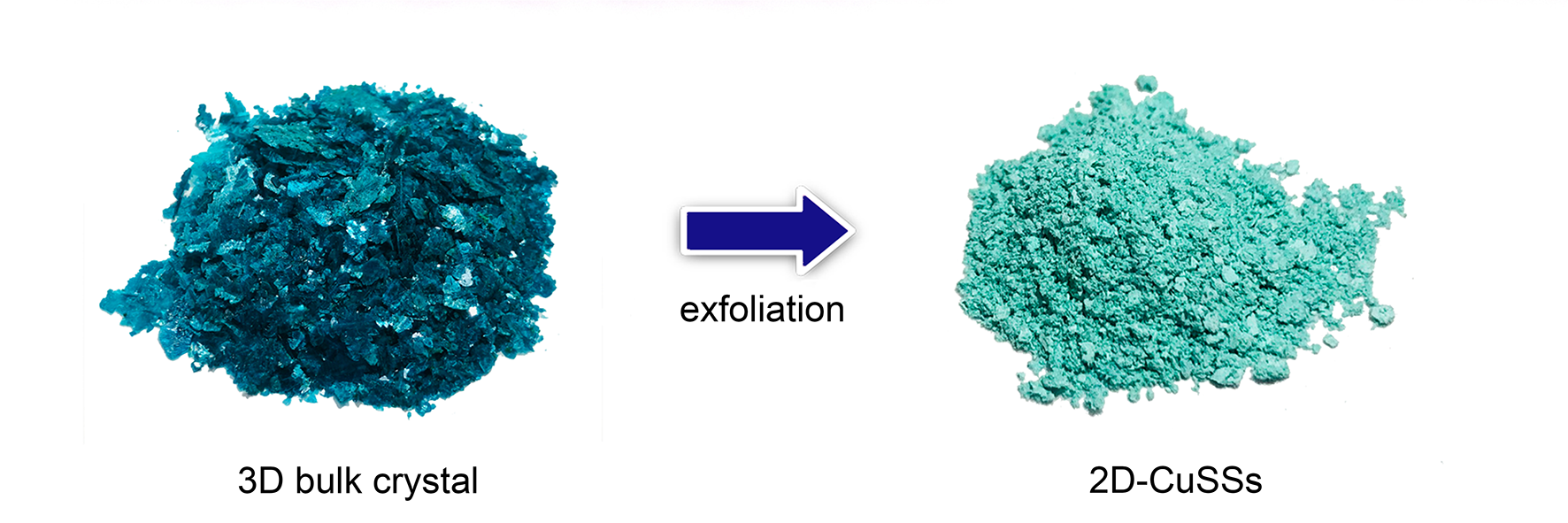


**Supplementary Fig. 3**: Photo of the bulk crystal (left) and 2D-CuSSs after exfoliation (right).


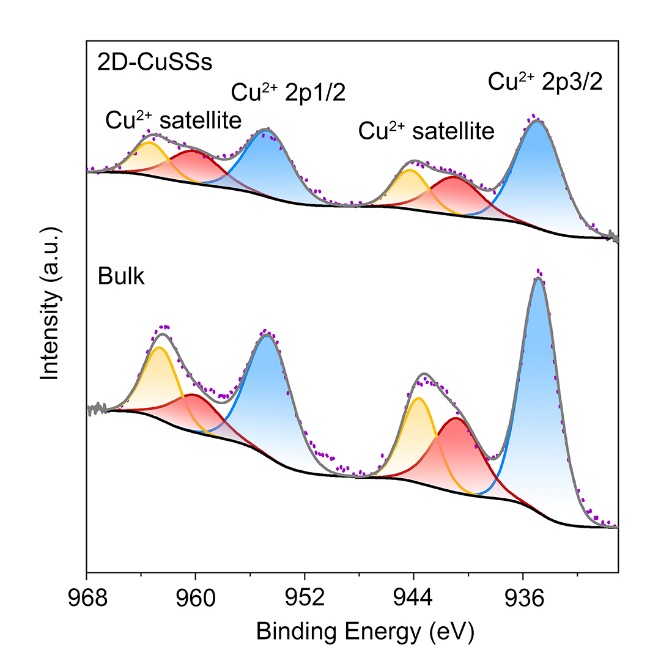


**Supplementary Fig. 4:** Cu 2*p* XPS spectra of bulk cuprate and exfoliated 2D-CuSSs. The features located at 934.8 eV and 954.8 eV with the corresponding satellite peaks indicate the valence state of Cu(II) of 2D-CuSSs.


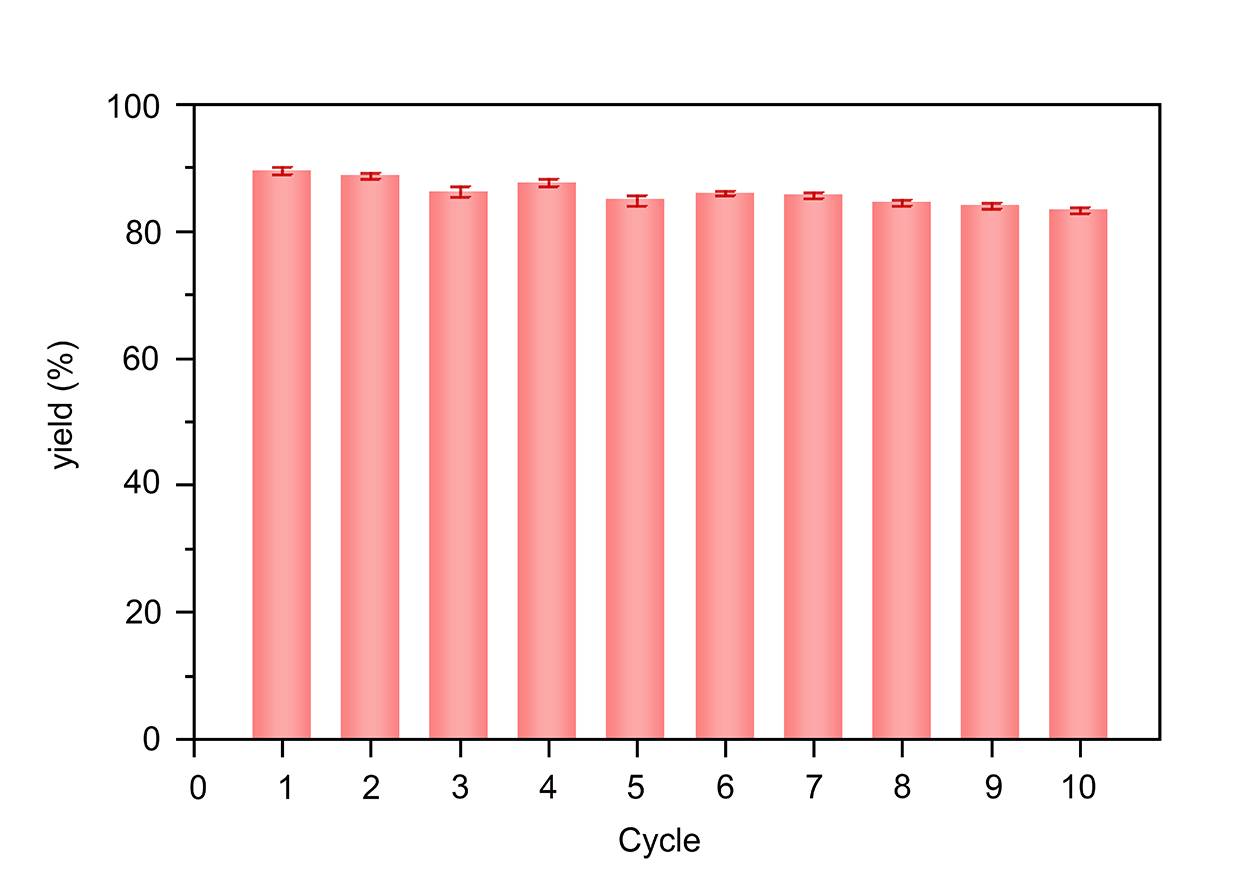


**Supplementary Fig. 5**: The recycle histogram for 2D-CuSSs for C-N coupling.


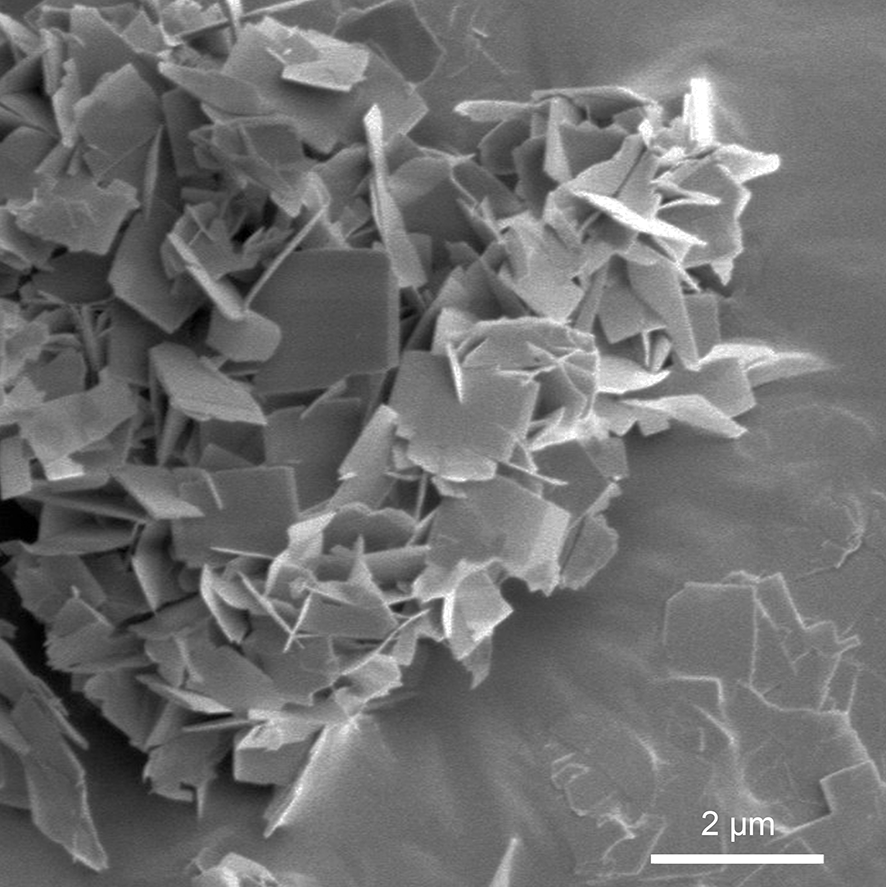


**Supplementary Fig. 6**: SEM image of the 2D-CuSSs after 10-time recycle.


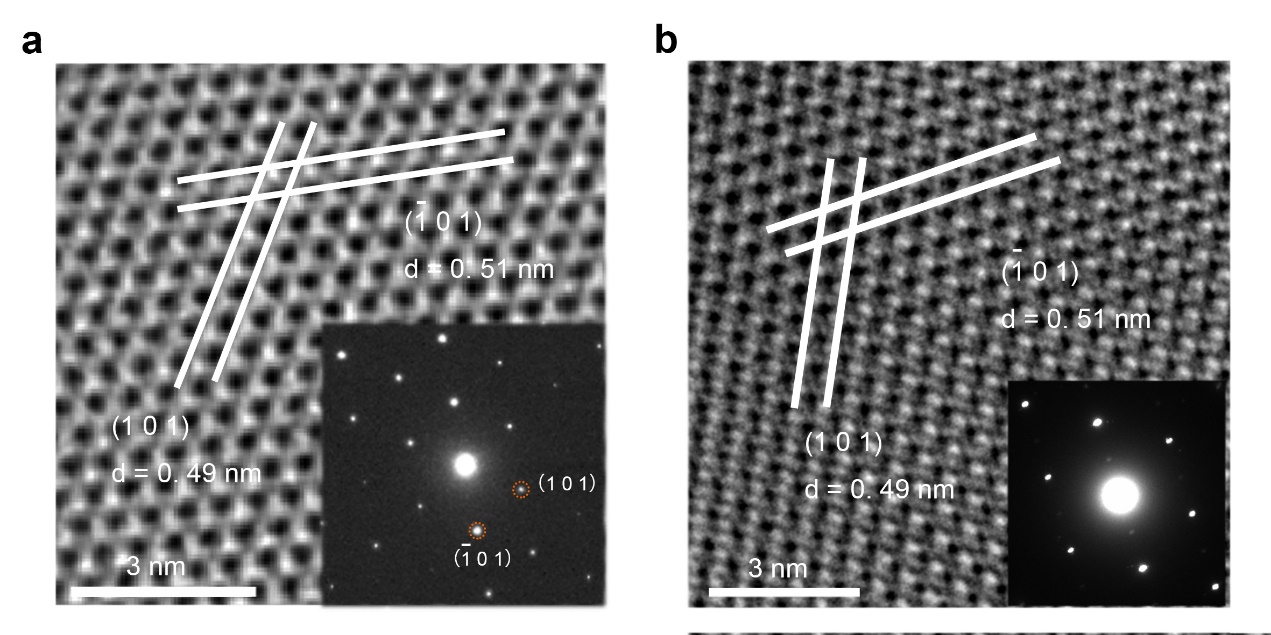


**Supplementary Fig. 7**: HRTEM image of the 2D-CuSSs before reaction (a) and after 10-time recycle experiment (b).

**Supplementary Fig. 8**: The scheme of the flow reaction with 2D-CuSSs.


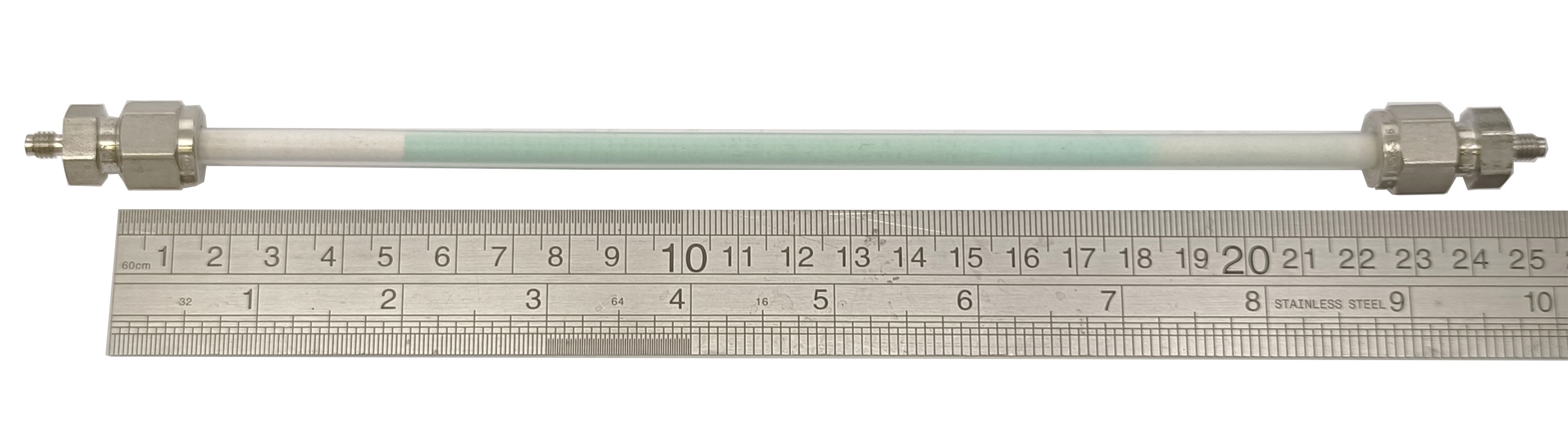


**Supplementary Fig. 9**: The flow reactor contains 2D-CuSSs sample.


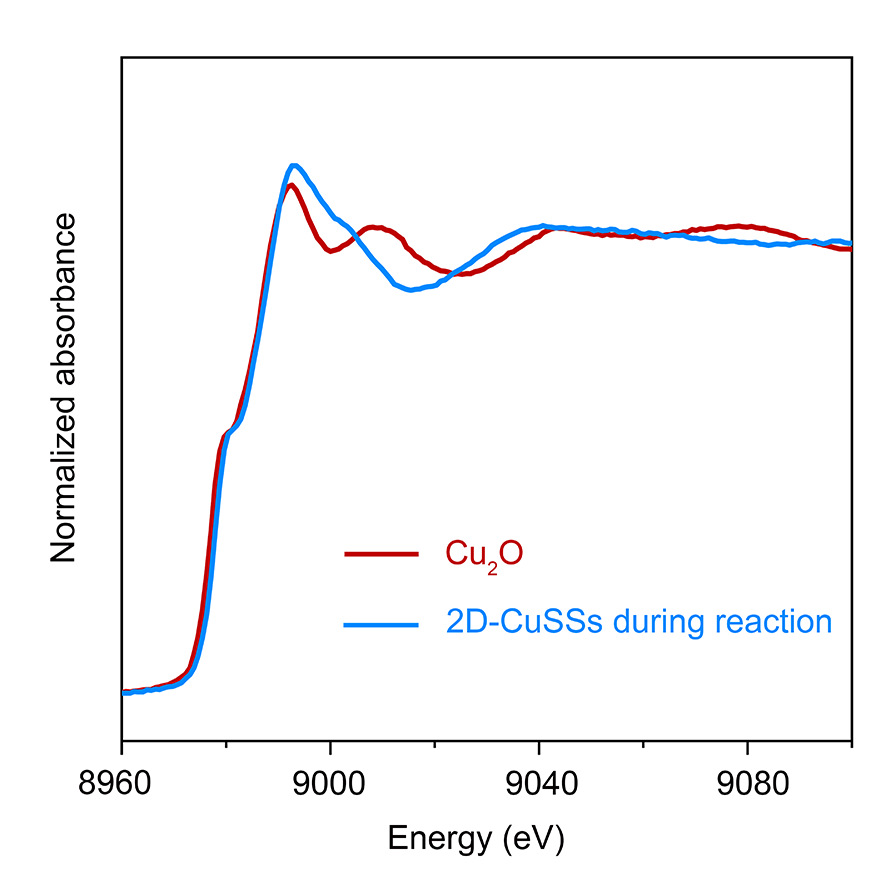


**Supplementary Fig. 10**: Cu K-edge XANES spectra of the 2D-CuSSs during the reaction compared with that of reference Cu_2_O sample.


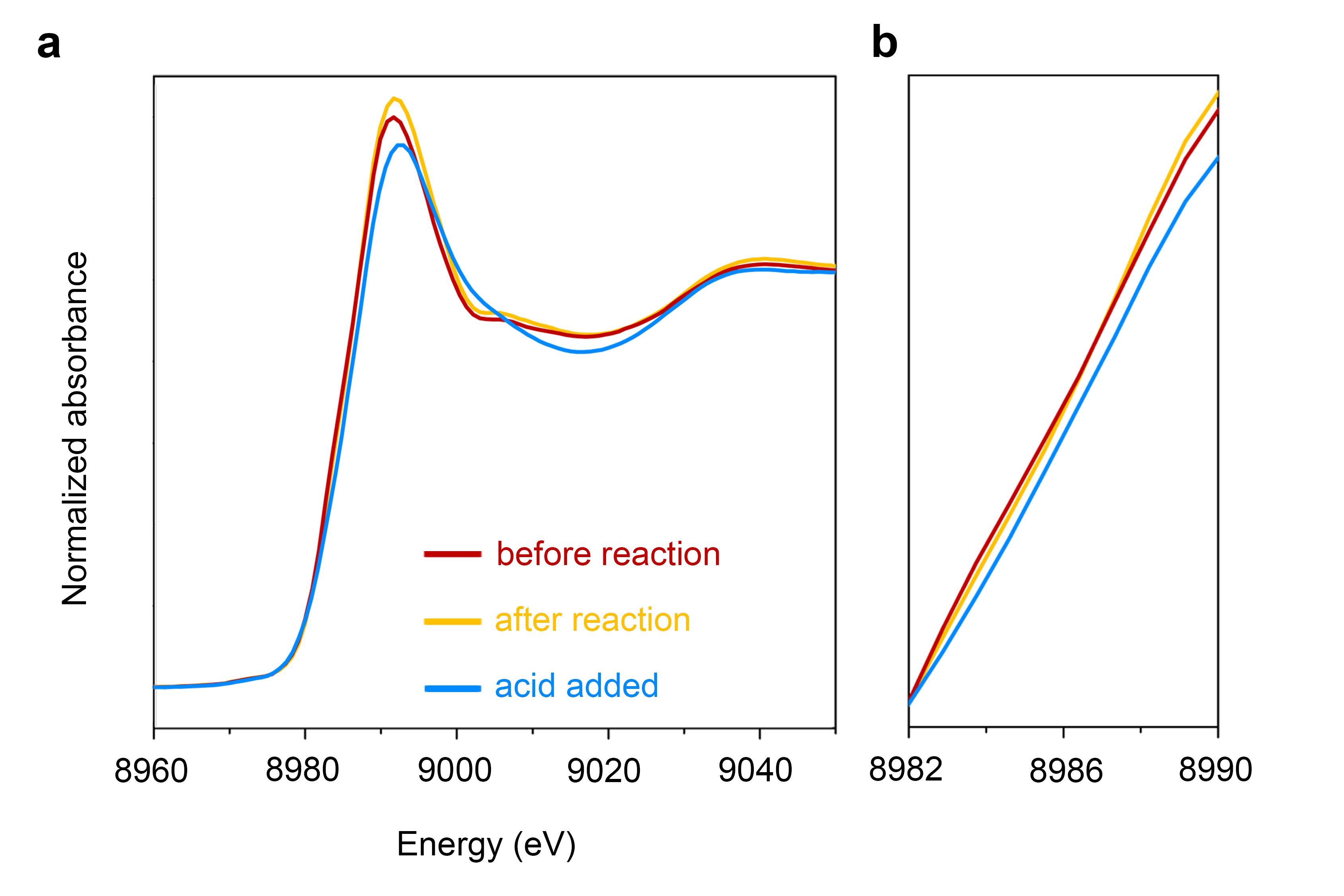


**Supplementary Fig. 11**: (a) Cu K-edge XANES spectra of the 2D-CuSSs before reaction, after reaction, and after acid treatment. (b) Magnified spectrum in a small energy window from 8982 eV to 8990 eV


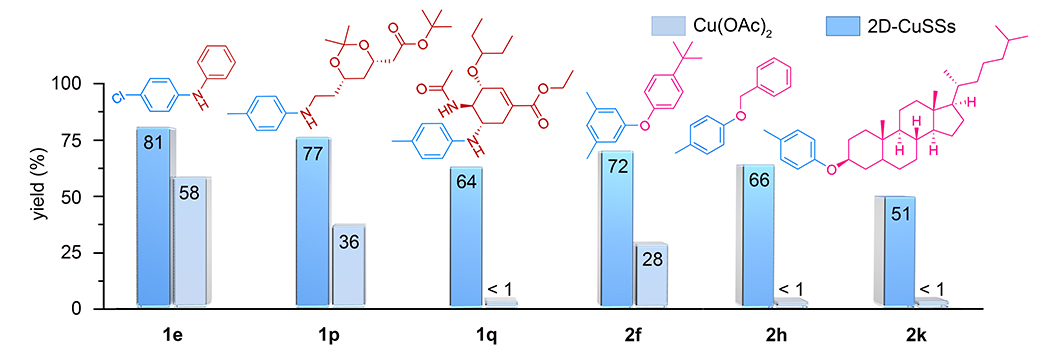


**Supplementary Fig. 12**: a direct comparison between 2D-CuSSs and Cu(OAc)_2_ for C-N coupling and C-O coupling.


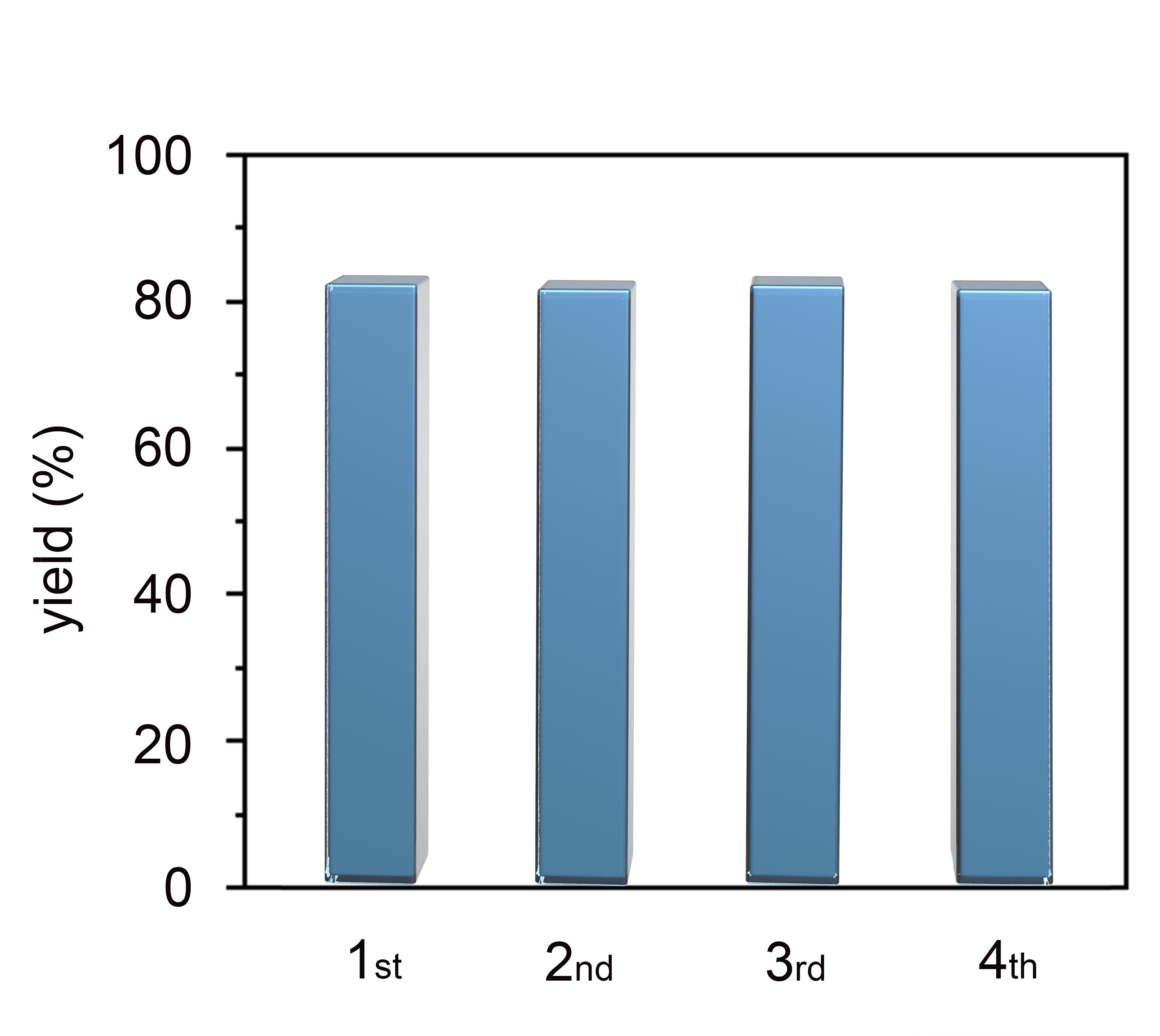


**Supplementary Fig. 13**: Continuous flow synthesis of 1i in a four-day period.


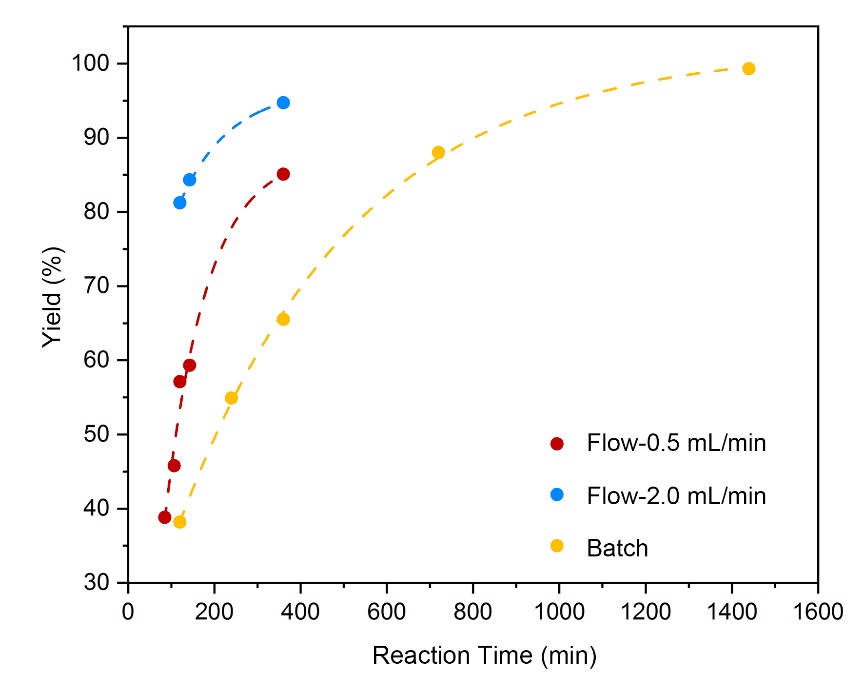


**Supplementary Fig. 14**: Comparison of continuous flow synthesis and batch reaction.

**Supplementary Table 1**. The comparison of catalytic performance under different conditions including different types of acids, different amount of acetic acid and catalysts for the C-N coupling.


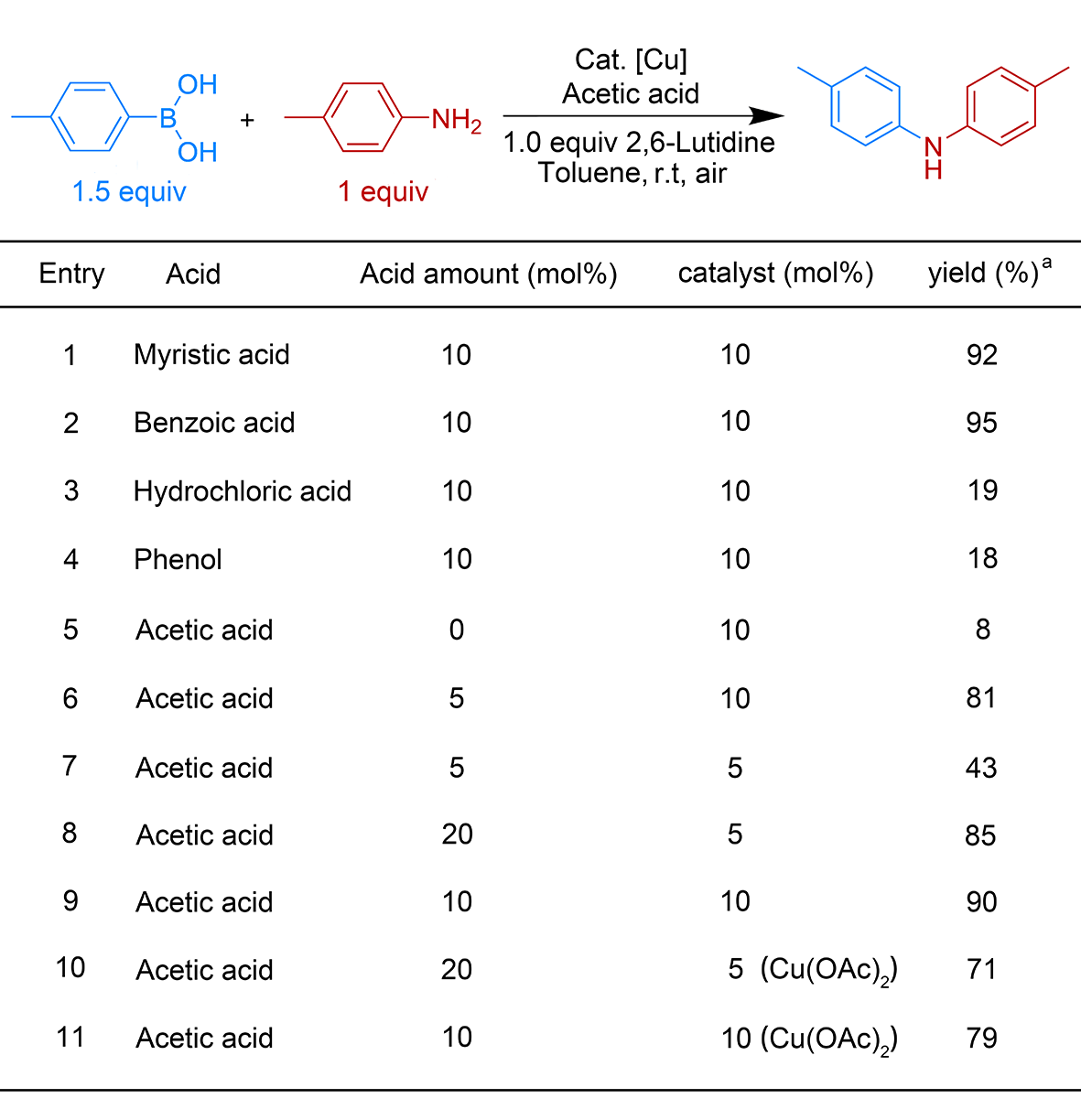


^a^4-Methylphenylboronic acid (0.375 mmol, 1.5 equiv), *p*-toluidine (0.25 mmol, 1 equiv), acetic acid (0.025 mmol, 0.1 equiv), 2,6-lutidine (0.25 mmol, 1 equiv), toluene (1 mL), room temperature, air, 24 h, isolated yield based on ^1^H NMR analysis.

**Supplementary Table 2**. Substrate scope of 2D-CuSSs for C-O coupling.


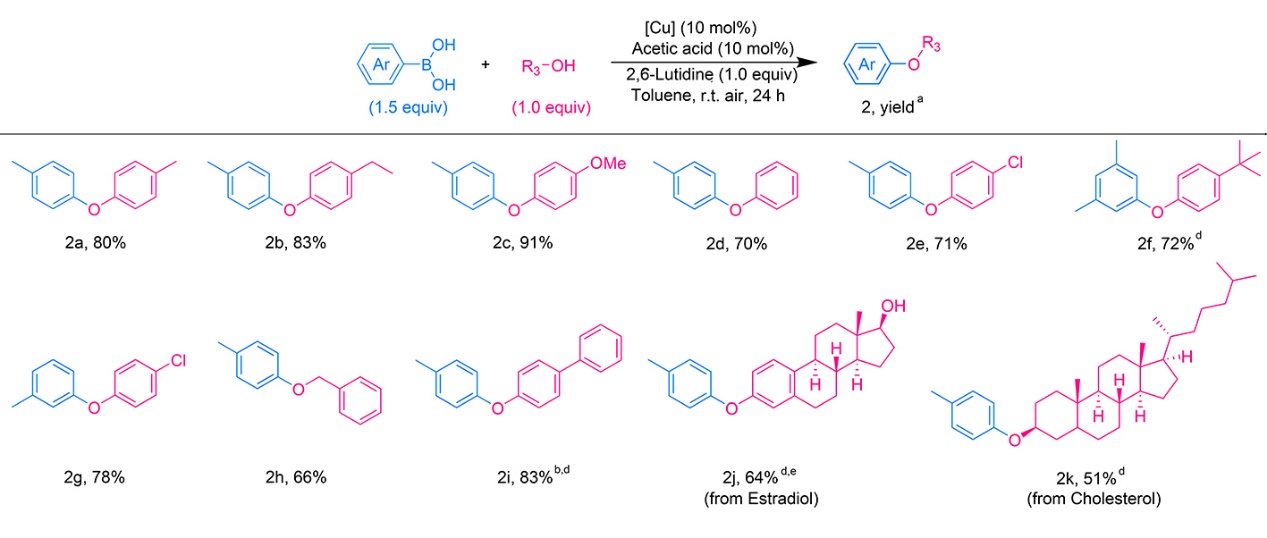


Reaction conditions: aryl boronic acid (0.375 mmol, 1.5equiv), amine or phenol (0.25 mmol, 1.0 equiv), catalyst (0.025 mmol), acetic acid (0.025mmol), 2,6-lutidine (0.25 mmol, 1.0 equiv), toluene (1 mL), room temperature, 24 h. ^a^Yield of corresponding amine and ether after reaction. Isolated yield. ^b^CH_3_CN (1 mL) was used. ^c^0.75 mmol boronic acid (3 equiv). ^d^20% catalyst. ^e^40 ºC.

**Supplementary Table 3**. Comparison of the stability with heterogeneous catalysts reported in the literature.


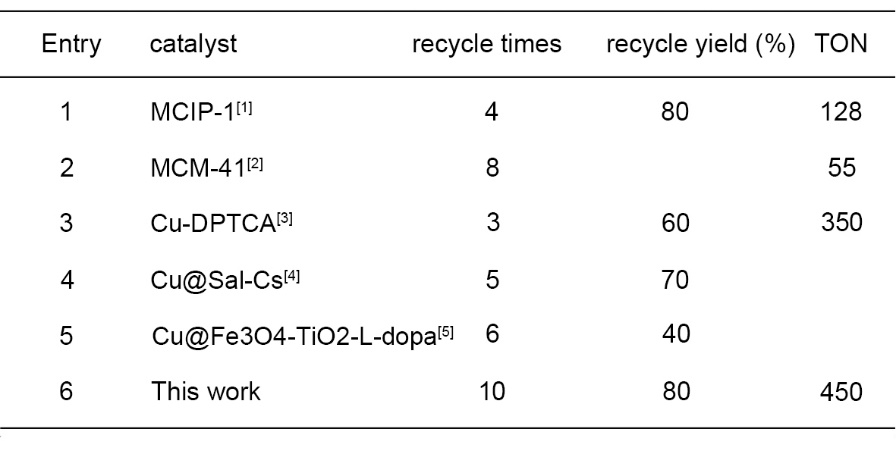


**Supplementary Table 4**. Optimization of reaction conditions with various copper-based catalysts.


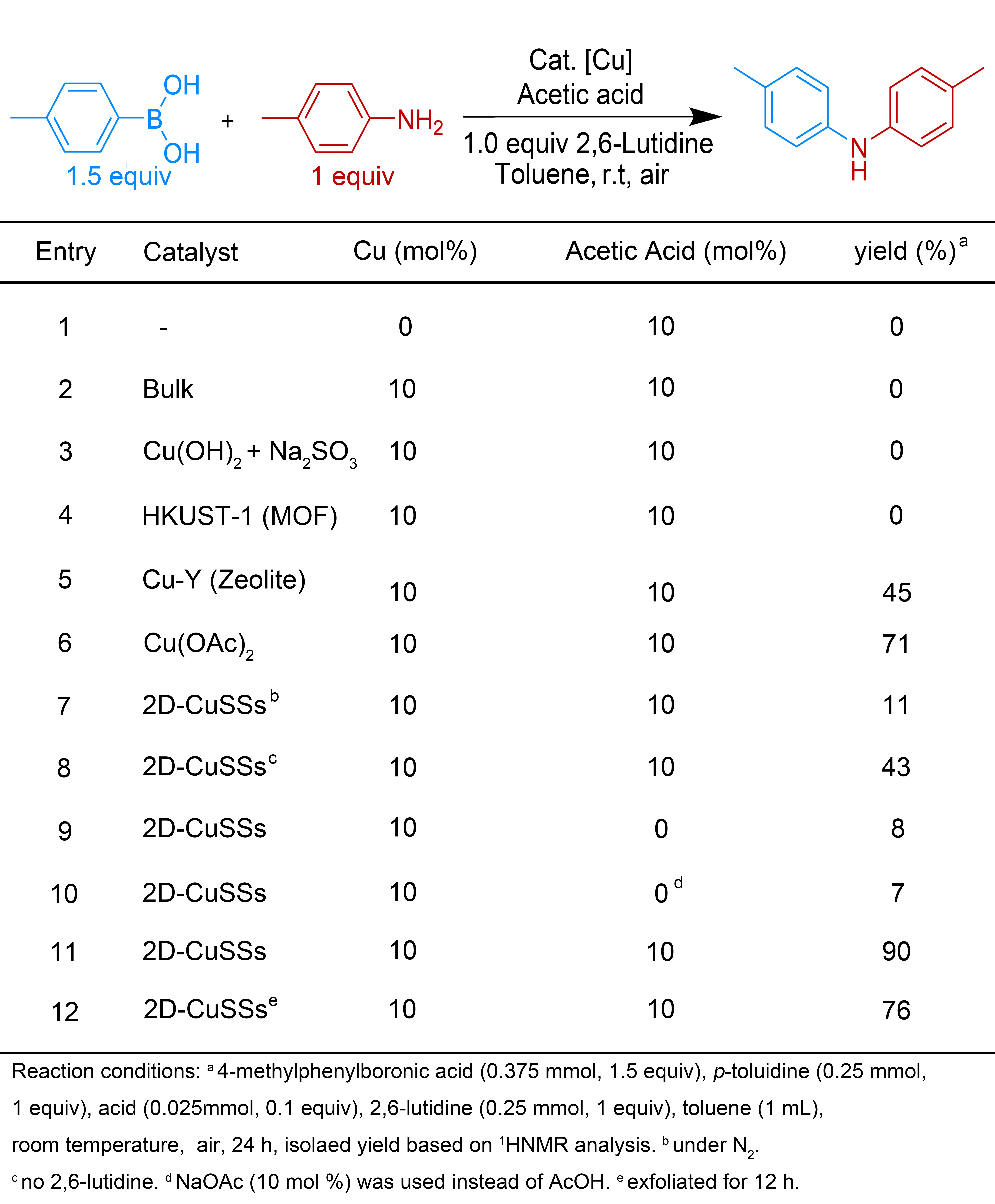


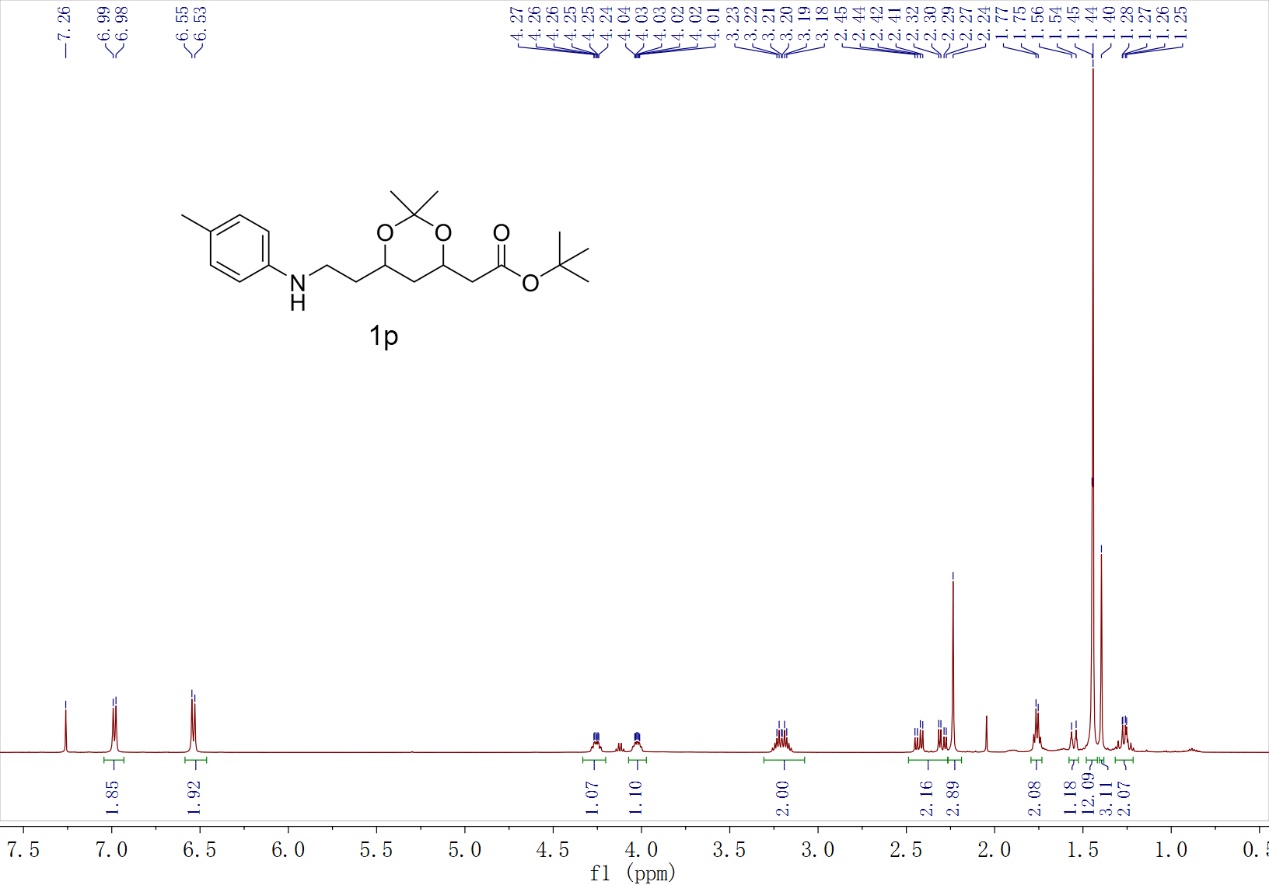

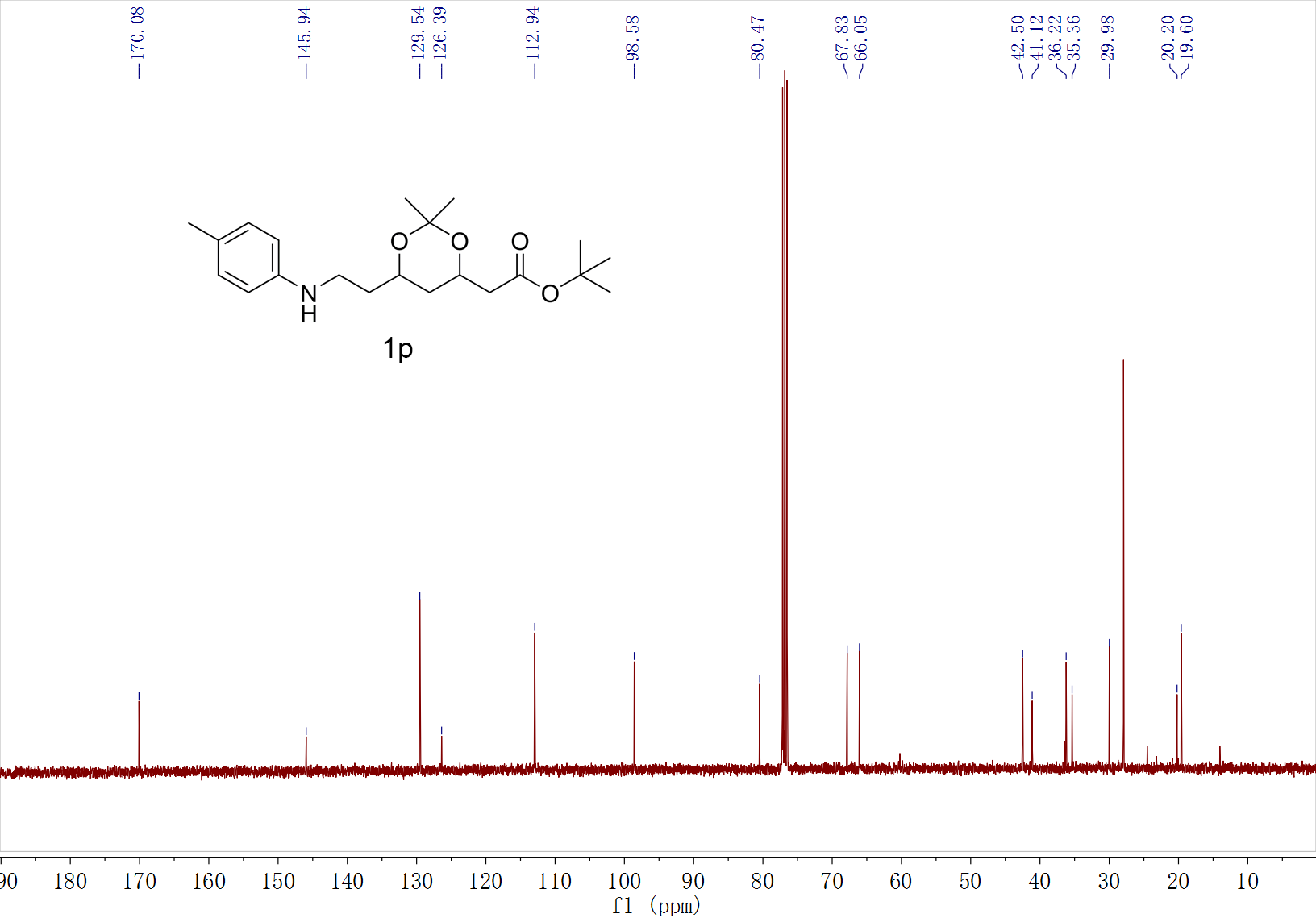
**Supplementary Fig. 15**: 1H and 13C NMR analysis for substrate **1p**.


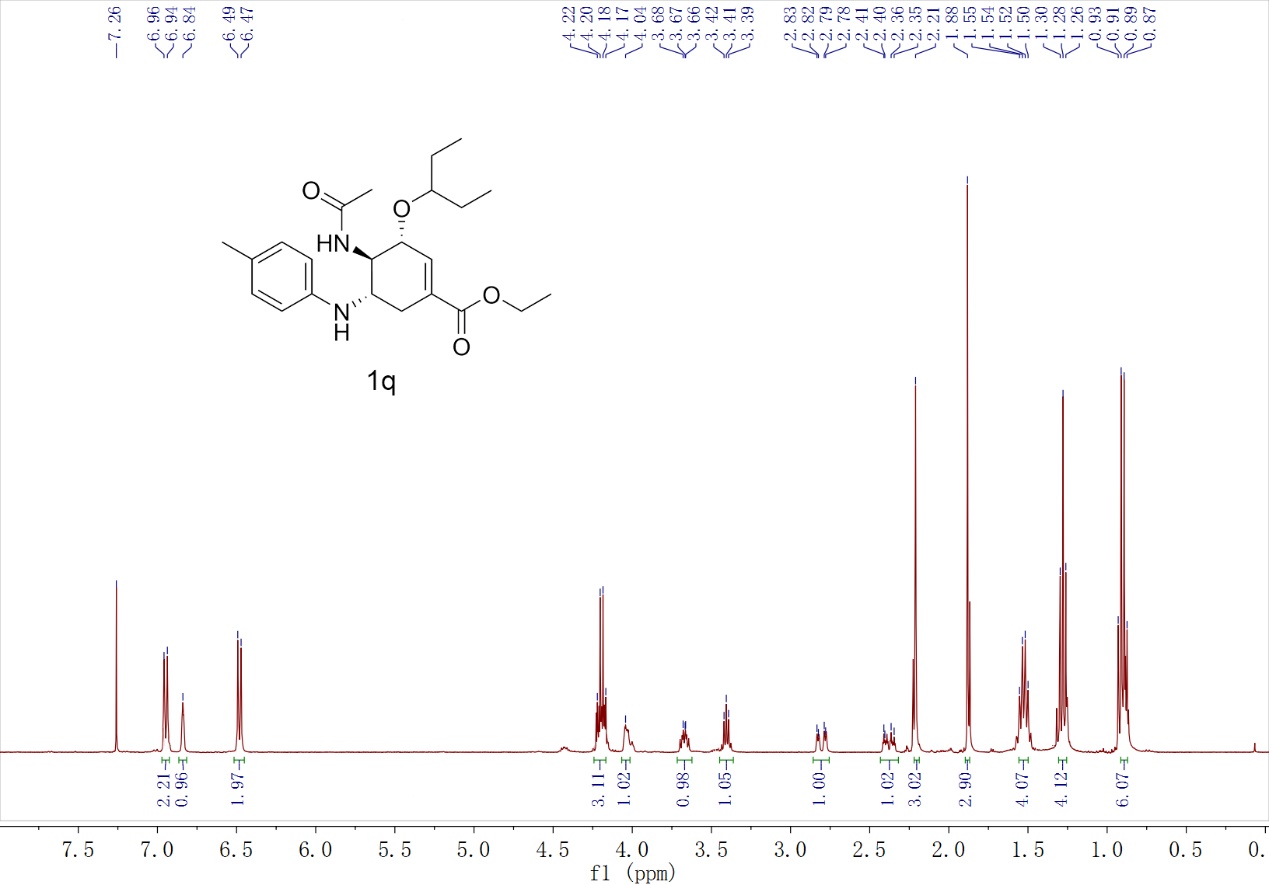

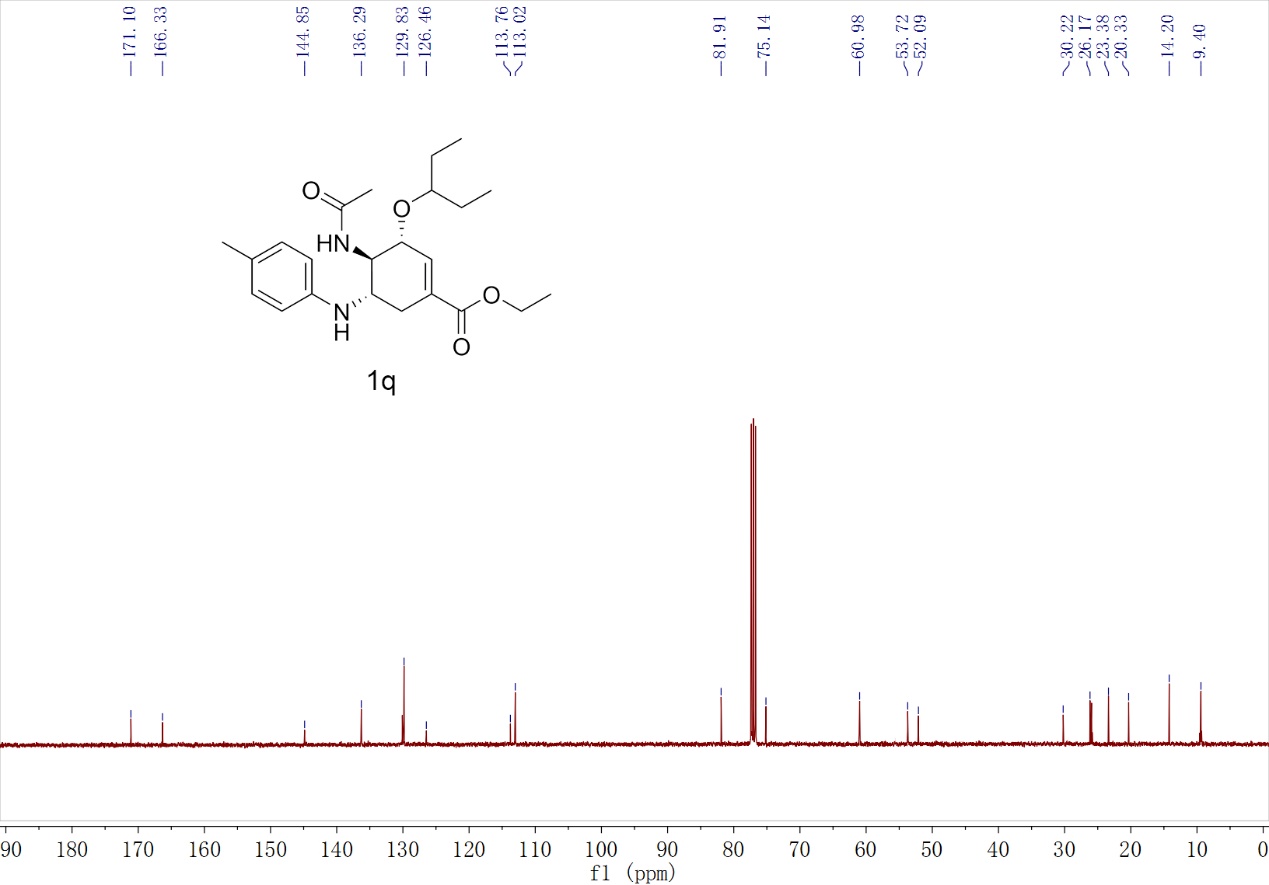
**Supplementary Fig. 16**: 1H and 13C NMR analysis for substrate **1q**.


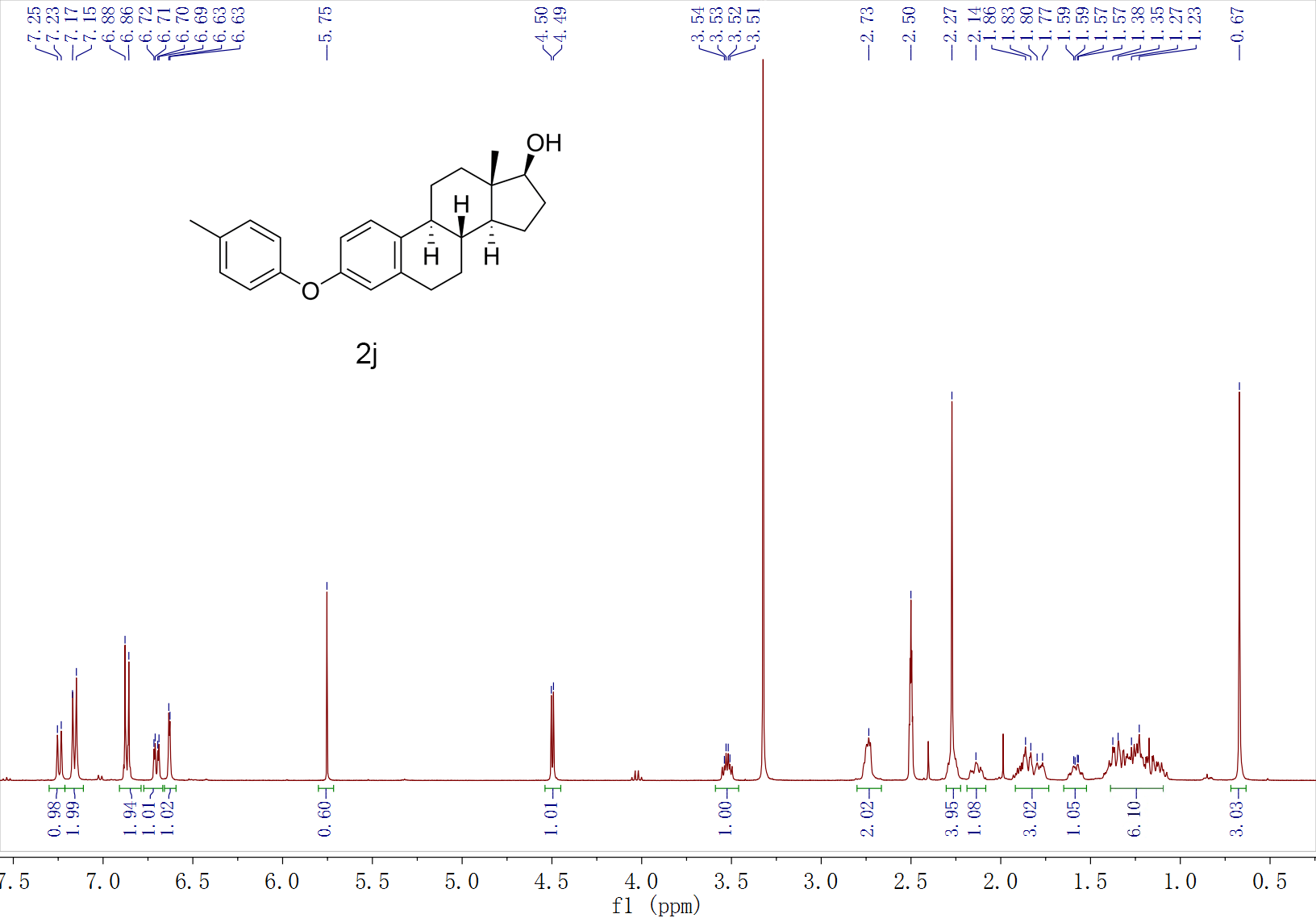

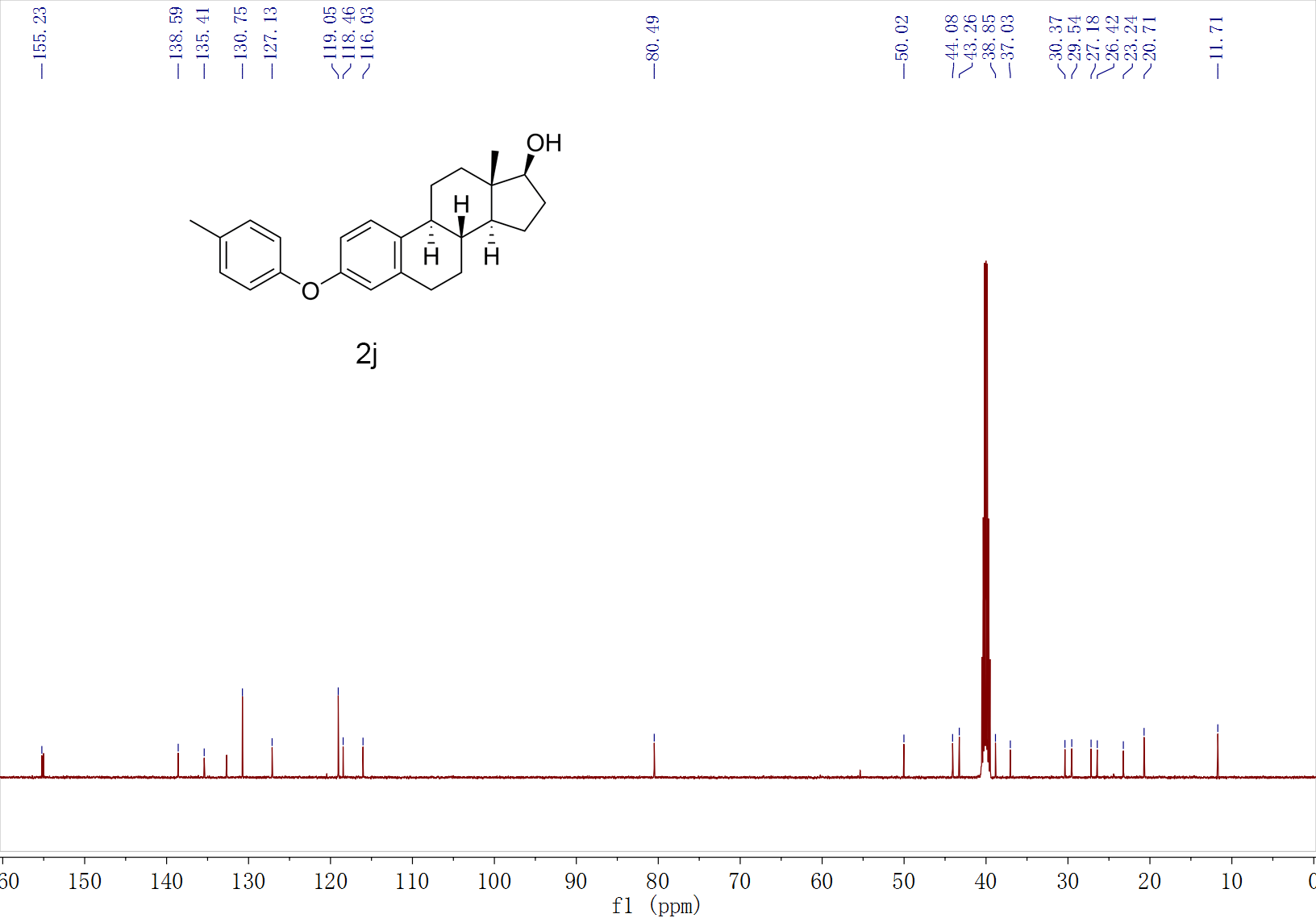
**Supplementary Fig. 17**: 1H and 13C NMR analysis for substrate **2j.
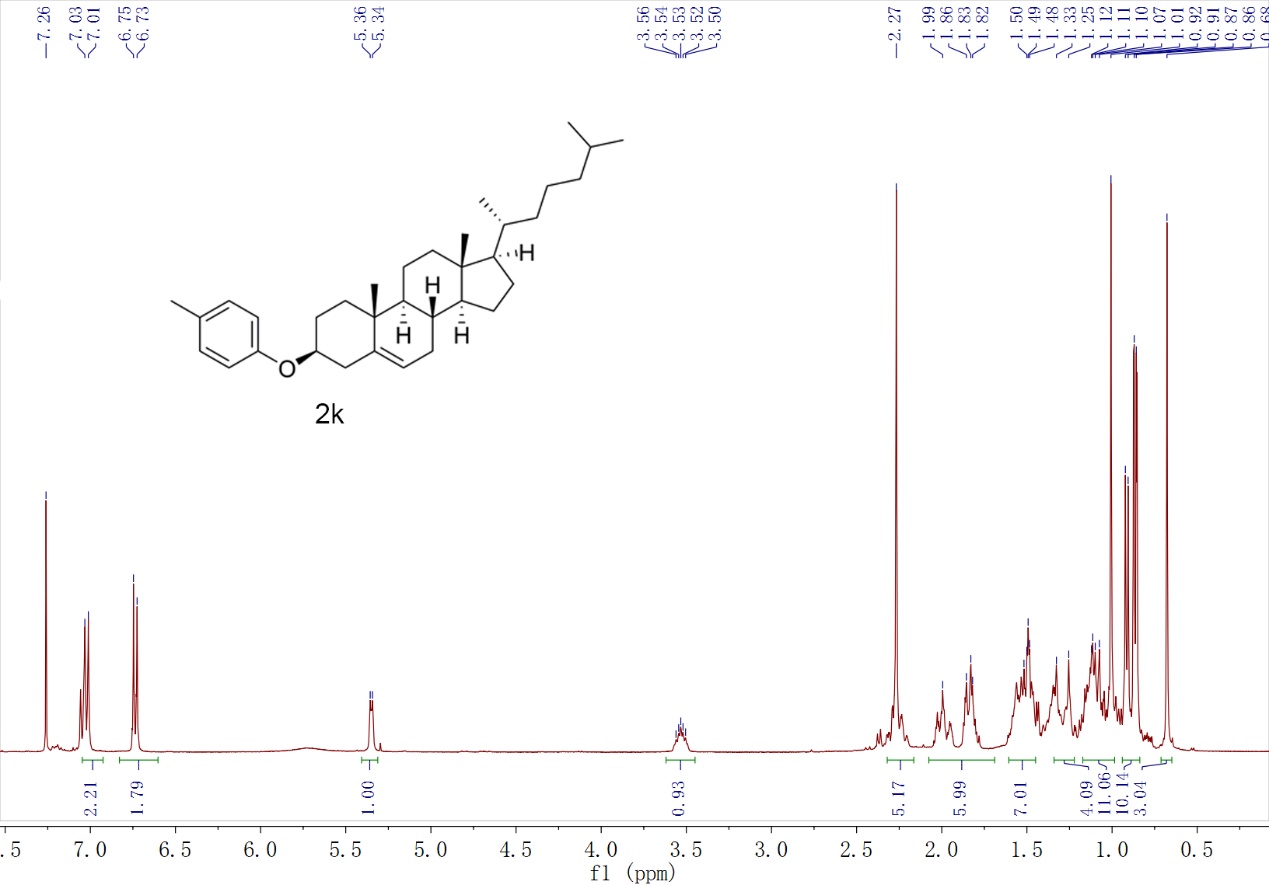

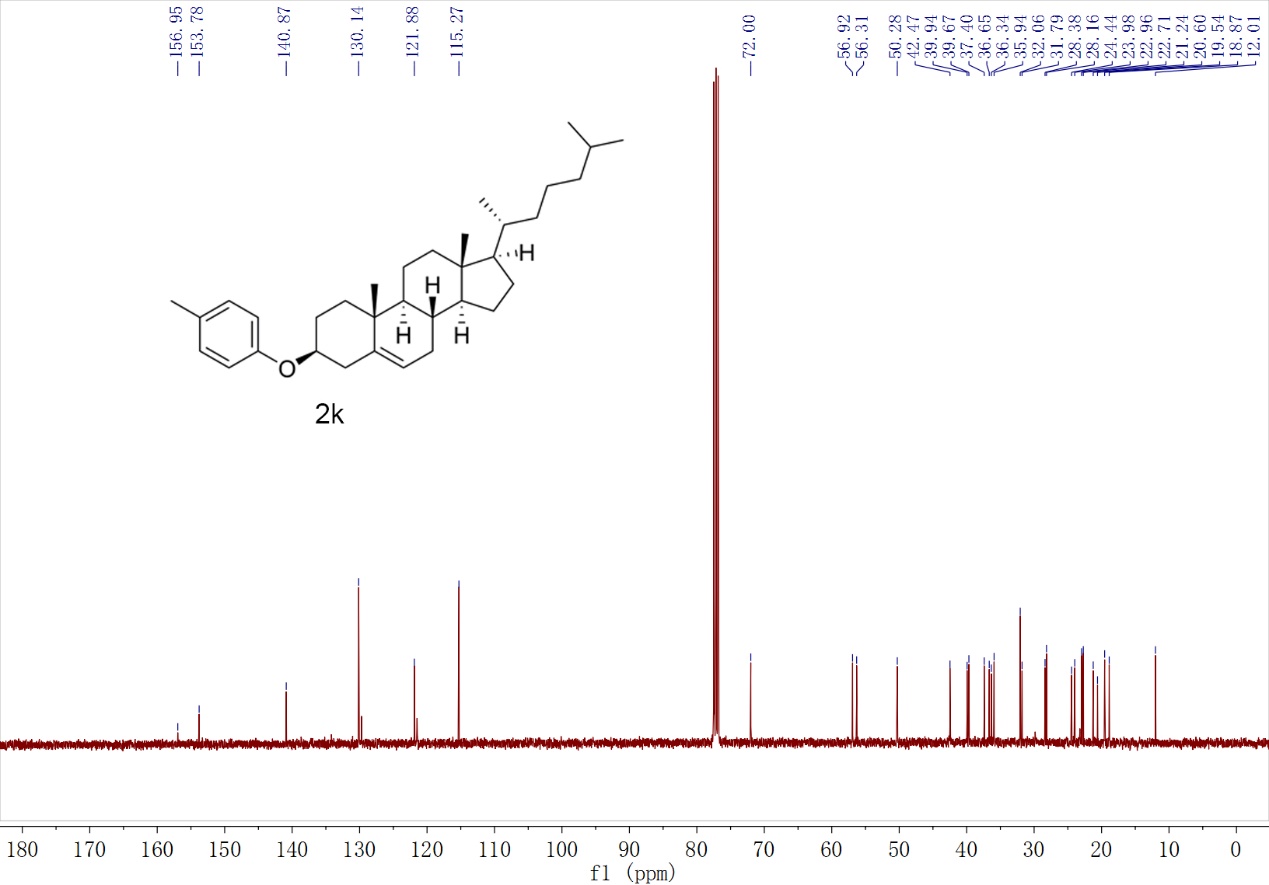
**

**Supplementary Fig. 18**: 1H and 13C NMR analysis for substrate **2k**.


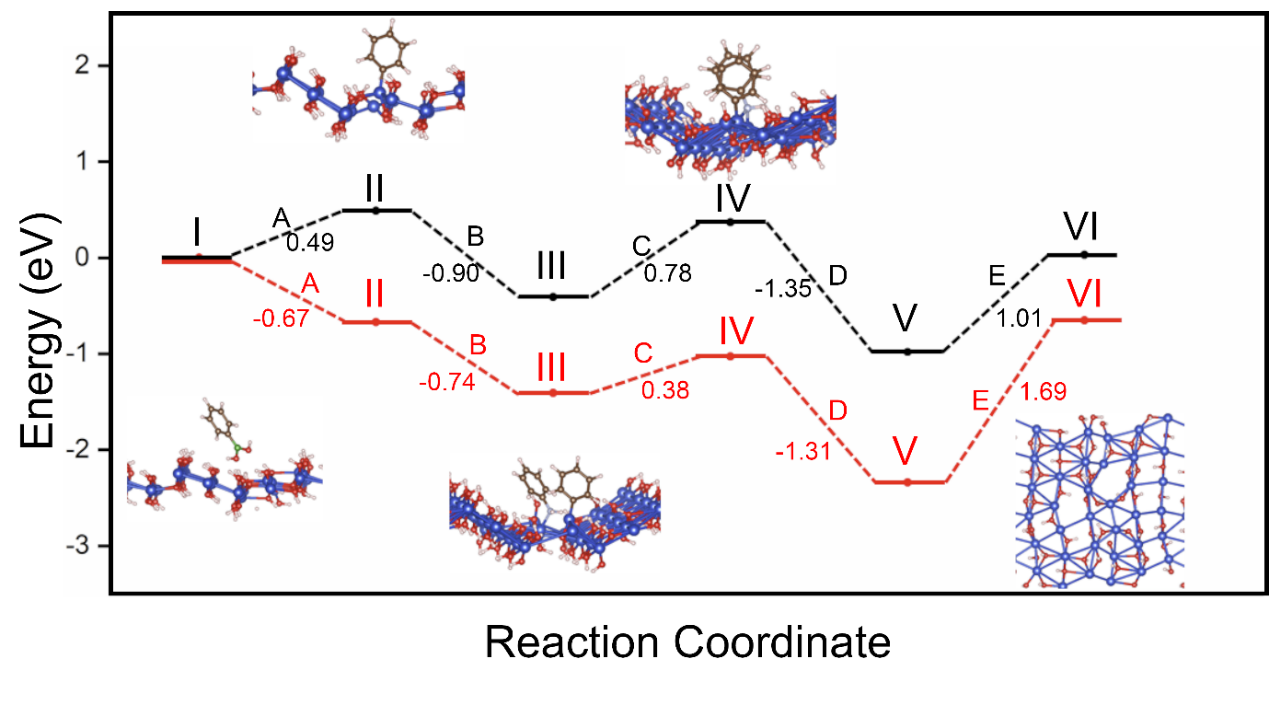


**Supplementary Fig. 19**: The calculated reaction energy diagram with the corresponding geometries: black line represents reaction thermal energy and red line represents reaction Gibbs free energy.


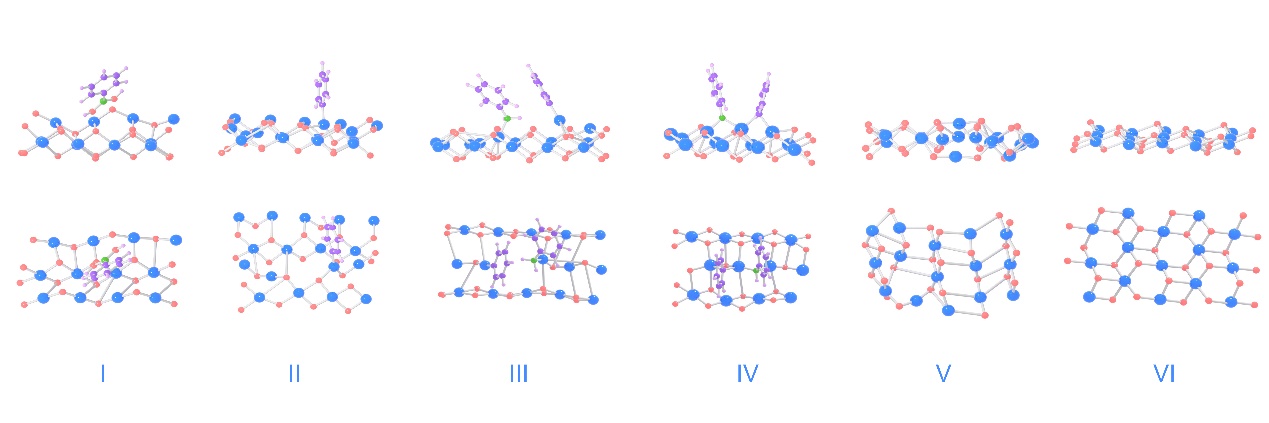


**Supplementary Fig. 20**: The side view and top view for six intermediates.

References:

1. Puthiaraj P, Pitchumani K. *Chem Eur J* 2014, **20**: 8761-8770..
2. Lin Y, Cai M, Fang Z *et al.*, *Tetrahedron* 2016, **72**: 3335-3343.
3. Zhang X, Qin J, Ma R et al., *J Chem Res* 2021, **45**: 795-799.
4. Asadi M, Naimi-Jamal M R, Panahi L. *Sci Rep* **11**: 18105.
5. Sharma H, Mahajan H, Jamwal B et al., *Catal Commun* 2018, **107**: 68-73.
6. Kumari S, Pathak D D. *Tetrahedron Lett* 2015, **56**: 4135-4142.
